# RTK Inhibitors in Melanoma: From Bench to Bedside

**DOI:** 10.3390/cancers13071685

**Published:** 2021-04-02

**Authors:** Malak Sabbah, Ahmad Najem, Mohammad Krayem, Ahmad Awada, Fabrice Journe, Ghanem E. Ghanem

**Affiliations:** 1 Laboratory of Oncology and Experimental Surgery, Institut Jules Bordet, Université Libre de Bruxelles, 1000 Brussels, Belgium; malak.sabbah@ulb.be (M.S.); ahmad.najem@bordet.be (A.N.); mohammad.krayem@bordet.be (M.K.); fabrice.journe@bordet.be (F.J.); 2 Medical Oncolgy Clinic, Institut Jules Bordet, Université Libre de Bruxelles, 1000 Brussels, Belgium; ahmad.awada@bordet.be

**Keywords:** RTKs, RTK inhibitors, resistance, drug combination, melanoma, c-KIT, EGFR, HGFR (c-Met), VEGFR

## Abstract

**Simple Summary:**

Receptor tyrosine kinases (RTKs) have long been demonstrated to play key roles in melanoma development. RTK activation requires dimerization and intracellular tyrosine trans-phosphorylation leading to downstream signaling pathways activation. As RTKs show different structures, mechanism of activation could differ. In this review, we will discuss the structure and specific mechanism of activation of each RTK, and its alteration associated with stage of the disease. Additionally, we summarize the effect of RTK inhibitors tested in preclinical and clinical melanoma studies indicating the reason, the reported results, and the rational approaches for combination strategies based on RTK inhibition in melanoma.

**Abstract:**

MAPK (mitogen activated protein kinase) and PI3K/AKT (Phosphatidylinositol-3-Kinase and Protein Kinase B) pathways play a key role in melanoma progression and metastasis that are regulated by receptor tyrosine kinases (RTKs). Although RTKs are mutated in a small percentage of melanomas, several receptors were found up regulated/altered in various stages of melanoma initiation, progression, or metastasis. Targeting RTKs remains a significant challenge in melanoma, due to their variable expression across different melanoma stages of progression and among melanoma subtypes that consequently affect response to treatment and disease progression. In this review, we discuss in details the activation mechanism of several key RTKs: type III: c-KIT (mast/stem cell growth factor receptor); type I: EGFR (Epidermal growth factor receptor); type VIII: HGFR (hepatocyte growth factor receptor); type V: VEGFR (Vascular endothelial growth factor), structure variants, the function of their structural domains, and their alteration and its association with melanoma initiation and progression. Furthermore, several RTK inhibitors targeting the same receptor were tested alone or in combination with other therapies, yielding variable responses among different melanoma groups. Here, we classified RTK inhibitors by families and summarized all tested drugs in melanoma indicating the rationale behind the use of these drugs in each melanoma subgroups from preclinical studies to clinical trials with a specific focus on their purpose of treatment, resulted effect, and outcomes.

## 1. Introduction

Receptor tyrosine kinases (RTKs) are ubiquitous cell-surface receptors in mammalian cells, which transduce cellular environment and interaction signals across the plasma membrane to several intracellular signaling networks [1,2]. Receptor tyrosine kinases are divided into 20 subfamilies all sharing similar molecular architecture that consists of an extracellular ligand binding domain, a single transmembrane helix, and a cytoplasmic region composed of a juxta membrane, tyrosine kinase domain (TKD), and a C-terminal tail domain [3]. RTKs are generally activated by receptor-specific ligands (growth factors) through receptor dimerization. There are four modes of receptor dimerization which are specific to each RTK family. The first mode is dimerization, completely ligand mediated without any physical interaction between the extracellular regions of the RTKs, e.g., Trka and p73. The second mode is ligand mediated dimerization through physical interaction between receptor as ErbB family members (EGFR,HER2/ErbB2, HER3/ErbB3, and HER4/ErbB4). The third mode of dimerization is mediated by ligand dimers that bind to two receptors and then interact with each other across the dimer interface e.g., SCF/c-KIT. In addition to bivalent ligand binding and receptor physical interaction, the fourth mode of dimerization is mediated by accessory molecules, eg., HGFR, FGFR family [4]. Whether the “inactive” state is monomeric or oligomeric, receptor activation requires binding of growth factor ligands to extracellular regions to induce an “active” state by ligand dependent induced receptor dimerization/oligomerization [4]. For most RTKs, this activation results in conformational changes allowing a trans-autophosphorylation of their tyrosine kinase domain and the release of the cis-autoinhibition conformation [2]. Several studies aimed to understand the consequences of dimerization on the activation procedure, the intracellular conformational switch and signal transduction. As receptor tyrosine kinases are involved in mediating cell-to-cell communication and in controlling several signaling and biological functions, their dysregulation, and consequently, the aberrant activation of their downstream intracellular signaling pathways, lead to many human diseases, such as diabetes, inflammation, severe bone disorders, arteriosclerosis, angiogenesis, and cancer [2]. Genomic studies in almost all types of human tumors show aberrations in several RTKs associated with tumor development and progression such as EGFR, HER2/ErbB2, MET, etc. [4]. RTK dysregulation in different cancers could be led by one of four mechanisms: activating mutation, gene amplification, chromosomal rearrangements, and/or autocrine activation. These observations led to the development of a number of small molecule inhibitors targeting RTKs that were tested in preclinical and clinical studies, and some of them were approved in some tumors associated with RTK dysregulation. Malignant melanoma is one of the most aggressive skin cancers that can disseminate and metastasize from a local site tumor to multiple organs, including lung, liver, brain, bone, and lymph nodes [5]. One of the most significant successful clinical practices in melanoma is the targeted therapies for activating gene driver mutations [6]. For this reason, a melanoma genomic framework was set to help specific therapeutic decisions [7]. Cutaneous melanoma is divided into four genomic subtypes: BRAF, NRAS, NF1, and Triple-WT. Candidate driver events in Triple-WT melanomas include KIT mutations/amplifications and co-amplified RTKs such as PDGFRA and KDR (VEGFR2). BRAF mutation is the most common in melanoma and occurs in 40–60% of cases [8]. Targeted therapy with BRAF/MEK inhibitors with or without immune check-point inhibitors (ICI) showed significant long-term treatment benefit in BRAF V600-mutated melanoma patients [9]. NRAS-mutation occurs in 15–20% of melanomas, and the approved ideal treatment for NRAS-mutant melanoma remains unknown, although several combinations of MEK inhibitors with Phosphoinositide 3-kinases (PI3K)–AKT (protein kinase B) pathway inhibitors [10] or CDK4/6 inhibition are proposed and under clinical investigation [11]. Recently, new RAS inhibitors targeting different aspects of RAS biochemistry were developed and provide hope that RAS inhibitors will eventually be deployed in the clinic [12]. Patients with KIT-mutant melanoma showed limited response rate to KIT inhibitors and ICI [13]. The latter aberration is most common in mucosal melanoma that most often arises in the oral cavity, nose and paranasal sinuses, genital tract, and anorectal region. Where cutaneous melanoma is common in European population, mucosal melanoma accounts for less than 1%. In other populations such as Asians, cutaneous melanoma is very low, and mucosal melanoma can be as high as 25% [14]. Mucosal melanoma treatment is arduous, because it is generally detected at a more advanced stage and responds less often to immunotherapy, with a mutational burden lacking MAPK activating mutation as compared to cutaneous melanoma [15]. Commonly to cutaneous melanoma, mucosal mutations shows alterations in SF3B1, KIT, and NF1 and less frequently mutations in BRAF and NRAS [16,17].

## 2. RTK Activation and Alterations in Melanoma

In 2017, a large WGS analysis of melanoma reported a frequency of aberrations in RTK pathway of 42% in acral and mucosal melanoma, which is considerably high [18]. Kinase profiling conducted in a panel of melanoma cell strains showed activation of several receptors including TYRO3, AXL, MERTK, EPHB2, MET, IGF1R, EGFR, KIT, HER3, and HER4 [19]. More importantly, several RTKs were found associated with resistance mechanisms to MAPK inhibition, phenotype switching, metastasis, invasion, and relapses [20,21]. This provided opportunities for preclinical and clinical efforts to effectively target RTKs molecular aberrations. Indeed, several RTK inhibitors were evaluated in preclinical settings and in clinical approaches, but few of the selective/specific inhibitors have been approved for melanoma treatment or are being tested in a specific subset of patients.

Due to the large number of RTKs described and tested in melanoma for different purposes, in this review, we will discuss and compare preclinical to clinical studies in terms of the effect of different RTK inhibitors and their derivatives alone or in combination and the associated resistance mechanisms to the main treatment. We shall also classify the utility of each RTK inhibitor in a specific subset of melanoma patients, treatment regimens, and circumstances of combinations with other treatment modalities.

### 2.1. c-KIT (CD117)

**Figure 1:**
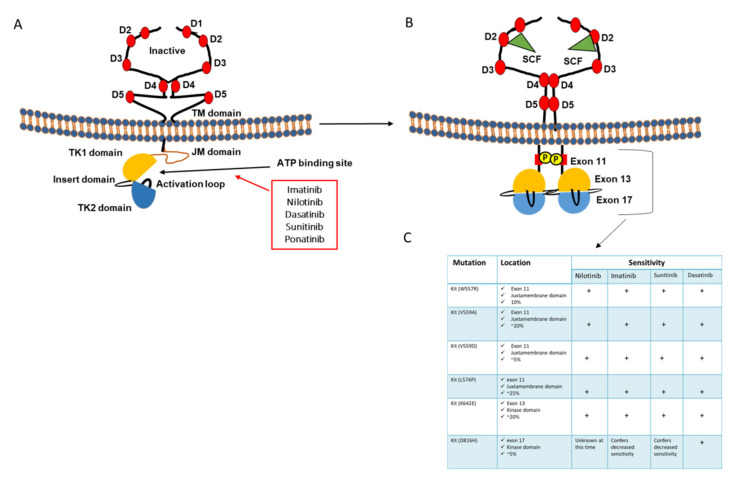
c-KIT structure, activation and mutations in melanoma. (**A**) The extracellular domain (ECD) of c-KIT consists of five Ig-like domains (D1–D5) followed by a transmembrane domain (TM), juxtamembrane domain (JM), tyrosine kinase 1 domain (TK1), insert domain, tyrosine kinase 2 domain (TK2), and activation loop domain. (**B**) Ligand homodimers bind to two c-KIT receptors, which interact with each other across the dimer interface. Dimerization mediates conformational changes and transphosphorylation of intracellular tyrosine residues. (**C**) Locations of most frequent c-KIT mutations in melanoma and its association with variable sensitivity to different RTK inhibitors.

c-KIT, a type III RTK located in a region on the long arm of chromosome 4 (4q11–4q13), encodes the stem cell factor (SCF) receptor (CD117) [22]. The type III class also includes the platelet-derived growth factor (PDGF) receptor (α- and β-chains), the macrophage colony-stimulating-factor receptor (CSF-1), and the Fl cytokine receptor (Flt3). Despite that, all RTKs shares the same topology, but what distinguishes the type III is the existence of five immunoglobulin-like domains in the extracellular region of the receptor (Figure 1A). The growth factor binds to the second and third immunoglobulin domains and the fourth domain is involved in receptor dimerization (Figure 1B). The KIT kinase insert domain at which tyrosine phosphorylation occurs and serves as docking site for downstream signal transduction proteins, is about 80 residues in length [23]. Stem cell factor binding to KIT leads to receptor dimerization that is mediated by both ligand homodimer (possibility of heterodimerization is still unknown) and a physical interaction between the two receptors, thus stimulating autophosphorylation in the kinase domain that activates the adaptor protein Grb2 (Tyr703), phosphatidylinositol 3-kinase (Tyr721), and phospholipase Cγ (Tyr730), leading to the stimulation of protein kinase activity. In addition, other autophoshphorylation in residues in the distal kinase domain can occur that attract other adaptor proteins such Crk (Tyr 900) and APS, Grb2, and Grb7 (Tyr936) [24]. The above listed interactions lead to the activation of several KIT downstream effectors such as AKT, Ras/MAPK, and JAK/STAT [25]. Residues 582–671 cover the small N-terminal lobe of the kinase (anchor and orient ATP), and residues 678–953 make up the large C-terminal lobe (which binds the substrate proteins) with a segment (where lies the KIT catalytic site) between them. The movement of the two lobes is relative to each other, and adopts simultaneous range of orientations, opening or closing the active site cleft) and is mandatory for the kinase activity of KIT. The open form allows ATP access and ADP release from the active site successively, and the closed form brings residues into the catalytically active state [23]. Disruption of this movement is mediated by the juxtamembrane (JM) region (residues 544–581), that favors the inhibition of the kinase activity. Particularly, a phosphorylation of tyrosine residue in the activation loop domain within the large lobe (begins with DFG (810–812) and ends with APE (837–839)) stabilizes the active conformation [23]. To summarize, stem cell factor binding induces RTK dimerization and favors transphosphorylation of two tyrosine residues (568 and 570) in the auto inhibitory JM domain resulting in the movement of the two lobes leading to a conformation conversion of the activation loop domain from a packed to an extended form.

Gain-of-function mutations as well as autocrine or paracrine activation of KIT have been described in several malignancies and may occur in exons that occupy the extracellular, JM, proximal and distal protein kinase domains. Due to distinct functions of receptor domains, all related activating mutations share the same ligand independent receptor activation but not the same structural rearrangement that is of particular importance for therapy. Gain of function mutations of c-KIT can be found in GIST (>90%), mast cell tumor (>70%) [26] and acute myeloid leukemia (>68%) [27]. Melanoma Triple-WT subgroup show the highest median KIT protein abundance with enrichment of KIT mutations, focal amplifications and complex structural rearrangements [7]. c-KIT activating mutations allow a ligand-independent activation of the receptor and consequently the constitutive downstream activation of MAPK, PI3K, Janus kinase (JAK)/Signal Transducer, and Activator of Transcription (STAT) [28]. As such, the use of drugs targeting c-KIT has provided a novel approach for cancer treatment. However, several issues have been raised regarding the development of c-KIT inhibitors. KIT mutation occurs in 25% of acral melanoma, 22% of mucosal melanoma, and 8% of cutaneous melanomas. Particularly, 81% of KIT mutations are distributed between exons 11 and 18 which include L576P, K642E, V559A, and D820Y mutations [29]. However, about 70% of c-KIT mutations occur in exon 11, with preponderance of L576P that shows poor sensitivity to imatinib in GIST [30], but variable sensitivities in melanoma [31,32]. Several other c-KIT inhibitors, including sunitinib, dasatinib, pexidartinib, sorafenib, ponatinib, and nilotinib, were tested and showed variable activity in c-KIT mutated melanoma as well. As mentioned above, KIT intracellular domains do not share one same function; consequently, activating mutation in juxtamembrane domain (exons 11 to 13) results in stabilization of the inactive conformation of the receptor despite its activation. However, an activating mutation in the activating loop domain (exons 17–18) results in continuous activation of the receptor showing stable active conformation. More importantly, RTK inhibitors show different binding affinities and most of them are unable to bind to the active conformation of the receptor, thus resulting in different treatment sensitivities. This is why we will discuss, within the present review, the effect of all c-KIT inhibitors tested in melanoma both in pre-clinical and clinical studies, with a specific focus on their efficacy according to the location of each exon mutation.

#### 2.1.1. Imatinib (GLEEVEC^®^)

Imatinib is a 2-phenyl amino pyrimidine derivative and a potent and selective inhibitor of the protein tyrosine kinase ABL, BCR-ABL, PDGFRA, and c-KIT. The active sites of tyrosine kinases each have a binding site for ATP to favor its enzymatic activity after ligand binding (protein tyrosine phosphorylation). Imatinib, binds close to the ATP binding site, locking it in a closed or self-inhibited conformation thus blocking the enzymatic activity of the protein [33].

As RTKs stand at the apex of cellular signaling and are considered as key transduction molecules, they became targets of interest also for melanoma. Indeed, melanoma cells express c-KIT, PDGF-R, and Abl, and an autocrine growth loop mechanism has been described for the receptor–ligand interaction of PDGF-R/PDGF, as well as for c-KIT/SCF [34]. Consequently, it was suggested that inhibition of RTKs may limit melanoma survival and proliferation. At this time, imatinib mesylate (Glivec^®^, formerly STI571, Novartis, Basel, Switzerland), used with success in CML [35] and GIST [36], has been used in a mouse melanoma model. Despite an efficient inhibition of PDGF-R phosphorylation within the tumor, no effect was observed on its growth (Table 1) [37]. Conversely, another study reported growth inhibition of B16F10 mouse melanoma cells in vitro and in vivo (Table 1) [38], but this did not translate clinically, and no objective responses were observed with imatinib in advanced metastatic melanoma based on the relative expression densities of receptor tyrosine kinases c-KIT and PDGF-R (Table 2) [39]. As imatinib showed remarkable effective clinical responses in several cancers [36,40], and as the expression of its targets (c-KIT, PDGFR) has been validated in melanoma, it was also tested in phase II trial but showed limited clinical responses as a single agent, even in tumors with high PTKs expression (Table 2) [41]. It was concluded that its clinical success may depend on a more precise selection of patients with exploitable tumor targets or in combination with other agents (Table 2) [42]. In 2006, Curtin JA et al. reported KIT as an important oncogene in melanoma and proposed that imatinib can be beneficial in this significant group of patient [43]. Consequently, several in vitro studies focused on assessment of imatinib in c-KIt mutant melanoma and reported significant sensitivities (Table 1) [44]. Coherently, the NCT00470470 clinical trial showed significant clinical responses of imatinib in a subset of patients harboring KIT alterations with overall response rate of 16% (95% CI, 2–30%) (Table 2) [31]. In parallel, a phase II trial with imatinib in China demonstrated overall response rate to imatinib of 30.2% (95% CI, 16–44.4) (Table 2) [45]. In accordance, the NCT00424515 trial indicated that imatinib was effective only in tumors harboring KIT mutations, and not KIT amplification and one of the mechanisms of resistance to imatinib could be associated to NRAS mutations or KIT copy number gain (Table 2) [32]. Furthermore, a diversity of KIT mutations was shown in melanoma, some were sensitive to imatinib, while others were either imatinib-resistant or not studied yet. Particularly, it was reported that melanoma non-responders harbor specific c-KIT mutations known to confer imatinib resistance in GIST, such as D820Y, N822K, and A829P, while responders show mutation on exon 11 or exon 13 [31,32]. In 2014, a case report expanded the melanoma population that could benefit from imatinib to those with somatic exon 8 KIT mutations [46].

Based on these studies, exon locations of KIT mutation as well as the ratio of mutant to wild-type KIT alleles were considered as predictive markers for clinical response to imatinib. Particularly, L576P or K642E mutation were associated with better clinical outcomes compared to those with a V654A or D820Y mutation already reported with resistance to imatinib in GIST. Taken together, the identification of KIT mutation is mandatory for selection of patients that could benefit from imatinib [47].

As imatinib was tested in phase II trials in melanoma with no significant long-term responses due to intrinsic or acquired resistance, several combination strategies were proposed. A phase I/II trial tested the safety and efficacy of imatinib in combination with bevacizumab (highly selective VEGF-A, the isoform that binds VEGF receptor (VEGFR) 1 and VEGFR2) in patients with advanced melanoma to prevent angiogenesis. The combination was well tolerated but did not improve clinical response (Table 2) [48]. Of interest, imatinib was reported to inhibit immunosuppressive mechanisms and to favor antigen- presenting cells function [49]. Accordingly, it was predicted that imatinib could be a promising candidate to synergistically enhance antitumor T-cell activation provided by CTLA-4 blockade immunotherapy [50] but showed response in one KIT-mutated melanoma patient and further investigation are still ongoing (Table 2) [50]. A Phase I/II study to evaluate the safety and efficacy of the combination imatinib with temozolomide in patients with unresectable, stage III/IV melanomas was launched and reported a complete response in one of seven patients and progression in the remaining. No further informations was reported later about this combination [51]. In 2016, a clinical trial was set up in order to evaluate the clinical benefit of combining pembrolizumab and imatinib in patients with locally advanced/metastatic melanoma harboring c-KIT Mutation/Amplification (NCT02812693), but unfortunately, the trial was withdrawn due to poor accrual [52]. Interestingly, a case report in 2019 showed a good control of the disease over two years with the combination after the failure of anti-PD-1 monotherapy in a patient with double KIT mutations (V559 and N822I). This combination was associated with increased manageable toxicity and tumor control before progression [53]. Recent evidence, from retrospective studies published in 2019, indicates the efficacy and safety of imatinib compared to other RTKi tested in melanoma with an overall response rate (ORR) of 21.8%, in addition to an ongoing open clinical trial with estimated completion year in 2022 (Table 2) [54].

### 2.1.2. Nilotinib (TASIGNA^®^)

Since it has become evident that some c-KIT mutant melanoma patients do benefit from imatinib, but develop resistance, and others show intrinsic/innate resistance to the drug related to exon mutation position on c-KIT, a second-generation of tyrosine kinase inhibitors have been introduced, including nilotinib. Nilotinib binds to the kinase domain of ABL/BCR-ABL,DDR, KIT, PDGF, and several EPH receptor kinases and maintains potency against a range of exon 9, 11, and 13 KIT mutations [67]. Nilotinib is a type II inhibitor; it binds to lipophilic ATP pocket, with a 30-fold higher potency than imatinib. Preliminary clinical results, showed a promising and durable response, with nilotinib in c-KIT metastatic melanoma patients showing mutation in exon 11, but nilotinib anti-tumor activity in melanoma patients with KIT amplification was not clear at this time (Table 3) [68]. In 2013, Todd et al. reported that secondary c-KIT mutations can confer acquired resistance to imatinib and nilotinib in c-KIT mutant melanoma cells (M230 c-Kit ^L576P^) and suggested alternative RTK inhibitors or inhibitors targeting the MAPK and PI3K signaling cascades to overcome resistance (Table 1) [55]. Unfortunately, these results were not observed clinically and none of c-KIT mutant melanoma patients developed secondary c-KIT mutation following treatment with imatinib or nilotinib [69]. Particularly, in 2015, a phase II trial with nilotinib in c-KIT melanoma was launched for patients who experienced disease progression, innate resistance to a prior KIT inhibitor, and a cohort of patients with brain metastases. The first results showed clinical benefit of nilotinib in some patients with melanoma harboring KIT alterations previously treated with KIT inhibitor (imatinib) but its efficacy in brain metastases was limited and needs further investigation (Table 3) [69]. In accordance, a phase 2 clinical trial of nilotinib in Korea for KIT mutant/amplified melanoma patients (UN10-06) indicates the safety and efficacy of nilotinib without showing any outperformance over imatinib effect in terms of progression free and overall survival (Table 3) [70]. Particularly, among the seven responders, five showed KIT mutations on exon 11, 1 patient showed mutation on exon 17 and 1 had KIT amplification (Table 3) [70]. In 2017, end-point clinical trial indicates that nilotinib could be an additional treatment option for KIT-mutated advanced patients or for intolerant patients to imatinib and in contrast to what have been observed in CML patients, nilotinib did not show any better response compared to imatinib and further proposed to investigate the potential role of combining c-KIT inhibitor to immunotherapy as a next step (Table 3) [71]. One case report in 2017, underlined the benefit of imatinib in a patient showing c-KIT tumor progression following treatment with niltoinib and ipilimumab [72]. In accordance with previous reports on imatinib and nilotinib, a phase 2 clinical trial in the French Skin Cancer Network showed that response to nilotinib is restricted to patients harboring exon 11 or 13 mutations, in addition to other factors that could be taken into consideration, such as high expression of PDGFR, BCL-2, and MCL-1 but low cyclin-D1 (Table 1 and Table 3) [56]. In addition, the authors bring evidence of STAT3 pathway inhibition in nilotinib responders and provide a rationale for future research assessing STAT inhibitors in the treatment of KIT-mutated melanomas [56].

### 2.1.3. Dasatinib (Sprycel^®^)

Dasatinib is an orally available, multi-kinase inhibitor targeting BCR-ABL tyrosine kinase receptor, SRC, c-KIT and ephrin receptors. Dasatinib received FDA approval in 2006 [73]. As at this time, Src and Yes were detected up-regulated in melanoma compared to normal melanocytes and dasatinib showed promising preclinical outcomes in breast, pancreatic, and colon cancer, it was tested for its efficiency in melanoma. In 2008, Eustace et al. showed that Src inhibition by dasatinib induces growth impairment, inhibits invasion/migration, favors apoptosis/G1 arrest, and enhances response to chemotherapy (temozolomide) in melanoma lines (Table 1) [57]. Dasatinib was also reported for its capacity to inhibit EphA2, Src family kinase particularly, FAK and Crk-associated substrate that consequently abolishes migration and invasion without any significant effect on melanoma cell viability or proliferation (Table 1) [58]. In contrast, it was shown that dasatinib, as Src inhibitor, impaired growth of melanoma cell lines and synergized with cisplatin but not with temozolomide or paclitaxel (Table 1) [60]. Moreover, a combination of dasatinib and dacarbazine was tested and showed its safety (70 mg dasatinib PO b.i.d with dacarbazine 800 mg·m^−2^) with clinical benefit in seven out of 11 (54.5%) patients (Table 4) [74]. Additionally, Woodman et al. in 2009 characterized the first cell line with L576P c-KIT mutation, the most frequent c-Kit mutation in melanoma. Surprisingly, this cell line showed resistance to imatinib but sensitivity to dasatinib due to receptor conformational change related to L576P mutation that affected imatinib-KIT binding ability (Table 1) [59]. Furthermore, the first phase 2 clinical trial of dasatinib in melanoma showed minimal clinical outcome in advanced unselected melanoma patients, indicating the importance of identifying predictive biomarkers for future use of dasatinib alone or in combination [75]. Nevertheless, a preclinical study conducted in 2013 indicated that dasatinib can impair growth, proliferation and induce morphological differentiation in only primary melanoma cells attributed to its ability to suppress activated ERK nuclear translocation (Table 1) [61]. In 2014 and due to the variable response to dasatinib reported in preclinical and clinical studies, Eustace et al. tried to identify predictive biomarkers and could identify a group of melanoma with high SRC, ANXA1, CAV-1, and EphA2 expression, which are more likely to benefit from dasatinib (Table 1) [62]. Furthermore, and based on preclinical studies indicating superior activity of dasatinib among other RTKi to the most common mutation on exon 11 ^L576P^ KIT, a phase II Trial (E2607) assessed dasatinib in KIT positive melanoma patients but the trial closed early because of slow accrual and too modest responses. It was concluded that due to its efficacy and limited toxicity, imatinib remains the treatment of choice for patients with unresectable KIT+ melanoma (Table 4) [76]. However, the discrepancy between the favorable effects of dasatinib in preclinical and the seldom efficacy in some patients highlights the need for reliable biomarkers to predict response in melanoma. Accordingly, preclinical studies published in 2018 indicates that dasatinib and dacarbazine combination was not synergistic, but put forward that the level of phosphorylated p53 (S46) significantly decreased in dasatinib-responsive cell lines attributed to an effect on its target p38 MAPK, which phosphorylates p53 at S46 and thus favors p53 function as an apoptosis inducer. The study concluded that investigating dasatinib responsiveness markers is of importance when considering future clinical trials evaluating dasatinib and DNA genotoxic drugs combinations to promote p53-dependent apoptosis (Table 1) [77]. Recently, SIRT2 was identified as important regulator of melanoma cells functions, such as cell motility, proliferation, and particularly resistance to dasatinib in melanoma (Table 1) [63].

### 2.1.4. Sunitinib (SUTENT^®^)

Sunitinib is an oral multikinase inhibitor that targets the vascular endothelial growth factor receptor (VEGFR) (VEGFR-1, -2, and -3), platelet-derived growth factor receptor (PDGFR) alpha and beta, c-KIT, and FMS-like tyrosine kinase receptor 3 (FLT 3), with potent antiangiogenic and antitumor activity [78]. Sunitinib showed good clinical outcomes in gastrointestinal stromal tumors (GIST) or metastatic renal cell carcinoma (RCC) and received FDA approval for these indications in 2006 [79,80]. First, a preclinical study reported that sunitinib significantly reduces vessel density and induces tumor hypoxia in melanoma xenografts. The latter effect may be beneficial if used as neoadjuvant treatment with radiotherapy or chemotherapy (Table 1) [64]. Another study reported an effect of sunitinib on melanoma tumor microenvironment without affecting tumor size (Table 1) [65]. Indeed, sunitinib inhibited both mutant KIT and VEGF receptors which is advantageous compared to other RTK inhibitors [81]. The first trial aiming at evaluating sunitinib in melanoma patients with mutations, amplifications, or overexpression of KIT showed benefit and proposed further studies (Table 5) [81]. However, a multicenter phase II study did not correlate clinical response to the presence of KIT mutation and attributed it to sunitinib antiangiogenic effect (Table 5) [82]. Another multicenter phase 2 trial in patients with metastatic mucosal or acral melanoma indicates an absence of significant difference between patients with or without KIT mutation (Table 5) [83].

### 2.1.5. Pexidartinib (TURALIO™)

Pexidartinib (TURALIO™) is an orally administered small molecule tyrosine kinase inhibitor that targets the colony-stimulating factor 1 (CSF1) receptor, KIT and FMS-like tyrosine kinase 3, showing an internal tandem duplication mutation (FLT3-ITD) [84]. An active phase I/II study to determine pexidartinib safety, pharmacokinetics, and preliminary efficacy in unresectable or metastatic KIT-mutated melanoma was launched in 2015 but is not recruiting anymore. First results posted but not published yet indicate one partial response from the six melanoma patients receiving 1000 mg/day [85]. In addition, a single-arm phase II trial (PIANO; NCT02071940) of pexidartinib in advanced KIT-mutated acral and mucosal melanoma is currently ongoing in the UK [84].

### 2.1.6. Ponatinib (Iclusig^®^)

Ponatinib was initially designed to inhibit BCR-ABL; other studies show its ability to target other kinases such as FLT3, c-KIT, FGFR, VEGFR, PDGFR, and c-SRC; consequently, it was classified as a multi-TKI [86]. It is an FDA-approved drug for chronic myeloid leukemia (CML) that showed promising outcomes in KIT-mutant PDX melanomas by comparing ponatinib to other RTK inhibitors [66]. Ponatinib downregulates phosphorylation of key signaling pathway mediators, particularly KIT, AKT, and ERK in a dose-dependent manner in all KIT-mutated PDX models (Table 1). In addition, it showed higher affinity to KIT^D816V^, a mutation located in the activating loop domain not recognized by most of the other RTK inhibitors including imatinib [66].

### 2.1.7. Sorafenib-Nexavar^®^

Sorafenib is an oral drug originally designed to inhibit RAF serine/threonine kinases (RAF-1, wild-type BRAF, ^V600E^ BRAF), but later in vitro studies indicated its efficacy against several receptor tyrosine kinases associated with tumor angiogenesis, such as VEGFR-2, VEGFR-3, PDGFR-β, and progression such as c-KIT and FLT-3 [87]. A clinical trial aimed to investigate efficacy and safety of sorafenib monotherapy in patients with progressive advanced melanoma showed its safety but indicated its modest clinical effect [87].

#### 2.1.8. c-KIT Inhibitors and Future Perspectives

X-ray crystallography of c-KIT has revealed various active and inactive conformational states that affect interaction with RTK inhibitors [88]. The active conformations are characterized by certain states of the activation loop, phosphate-binding loop (P-loop), and helix C, which direct the catalytic machinery to phosphorylate substrates. In the inactive conformation, one or more of these elements are in others states, which does not allow substrate binding and/or catalysis [89]. Crystallographic studies have shown that imatinib binds the inactive conformation and KIT-imatinib interaction deviates from the auto inhibited inactive KIT kinase; this prevents inhibition of A-loop mutations, as it confers an active state of the kinase and confers decreased sensitivity to imatinib (Figure 1C). Despite that, a second generation of KIT inhibitors were developed and expected to be more efficient; as they bind to both active and inactive conformations of c-KIT, imatinib remains the c-KIT inhibitor of choice in comparison to other c-KIT inhibitors in melanoma. This is due to other constraints associated with other kit inhibitors such high clinical toxicity (sunitinib) or activation of other mechanisms that counteract drug clinical activity (dasatinib, nilotinib). The limited efficiency of imatinib and its derivatives indicates the importance of the development of new c-KIT inhibitors with specific consideration of the binding potential to both active and inactive conformations of the receptor.

### 2.2. EGFR

The epidermal growth factor receptor (EGFR) was the first receptor tyrosine kinase (RTK) discovered and is a type I glycoprotein located on chromosome 7p11–13 that includes ErbB2, ErbB3, and ErbB4 [90]. In humans, several ligands have been identified to bind to the EGFR family: EGF, TGFα, AREG, HB (heparin binding)-EGF and a number of virally-encoded factors [91]. Importantly, these ligands may activate different biological processes within the same cell [92]. For example, TGFα and AREG stimulate higher proliferation than do EGF and heparin [93].

**Figure 2:**
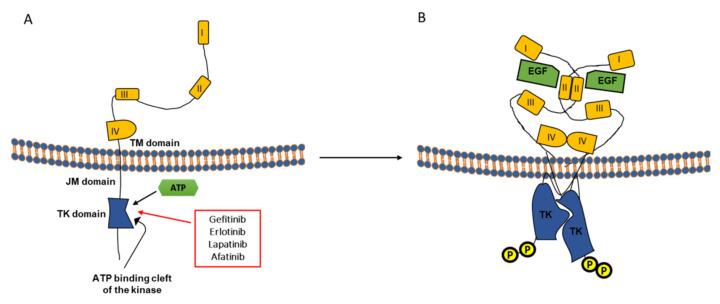
EGFR structure and activation. (**A**) ECD of EGFR consists of four subdomains noted, respectively, I, II, III, and IV, followed by a transmembrane domain (TM), juxtamembrane domain (JM), and tyrosine kinase domain (TK). (**B**) Ligand binds to domains I and III simultaneously, and mediates physical interaction between receptors. This favor intracellular conformational changes and kinase activation by allosteric mechanism. EGFR inhibitors are ATP competitor and act by binding the ATP binding cleft.

EGFR comprises an extracellular domain (ECD) composed of 620 residues, a kinase domain (residues 685–957) connected by a transmembrane helix (residues 621–642), and a short juxtamembrane segment [94]. This family is characterized by ligand binding to the glycosylated external domain composed of four subdomains designated domain I, II, III, and IV or L1, S1, L2, and S2, respectively (Figure 2A). The domains I and III form the ligand binding domain of EGFR, while other parts mediate receptor dimerization and interactions with other membrane proteins (Figure 2B). EGFR monomers predominate before ligand-binding, while after and like other RTKs, they undergo dimerization (homo/heterodimers), in a back-to-back orientation. In this second mode of dimerization, ligand mediates dimerization through physical interaction between receptors. In its inactive state (absence of ligand), the extracellular region adopts a “tethered” configuration consisting in a dimerization by a β-hairpin within subdomain II of the ECD, and interaction with domain IV to consequently form an intramolecular autoinhibitory conformation [4]. Ligands bind simultaneously to the two sites in ECD subdomains I and III, rather than binding two separate receptors as is the case with SCF (see above). This induces a dramatic conformational change, particularly an extension in the ECD to expose the buried dimerization arm (subdomain II and IV) in an active state [4] that consequently favors intracellular conformational changes and allows kinase activation (Figure 2B) [94]. The latter contributes to stabilizing extracellular contacts, allowing the movement of the transmembrane helices and a destabilization of the intracellular contacts between the C-terminal and the kinase domains. The active kinase form is mediated by allosteric mechanism rather than phosphorylation, by which the c-terminal lobe of one kinase forms an asymmetric dimer with the N-terminal lobe of the second kinase following dimerization and constitutes a complex known as CDK/cyclin-like complex [95,96]. Like other kinases, receptor dimerization results in a transphosphorylation of tyrosine residues in the C-terminal domain, which serve as docking sites for signaling molecules that contain SH2 or PTB domains to consequently activate signaling pathways [97]. Unlike most kinases, phosphorylation of the EGFR activation loop is not mandatory for its activation. Ligand binding favors a contact between the extracellular domains, causing a destabilization of the intermonomer contacts within the intracellular domain, causing its complete activation [98,99]. Thus, interaction between the intracellular domains regulates receptor activity [100]; particularly, the C-terminal domain acts as an inherent negative regulator [94]. Ligand-induced receptor internalization and degradation results in signal attenuation with net removal of either the receptor itself in the case of non-dissociative ligands like EGF, or the ligand for dissociative ligands such as TGFα [91]. Receptor endocytosis relies on specific adaptins and sorting nexins complexing with carboxy-terminal motifs, while the destiny of the receptor depends on its continuous occupancy and kinase activity.

Gain of function mutations and overexpression of this family of receptors were implicated in a variety of human malignancies, such as mammary carcinomas, squamous cell carcinomas and glioblastomas [2,101]. Due to the functional involvement of EGFR in diverse cellular mechanisms, several therapeutical strategies have been developed in various human malignancies with either the use of anti-receptor monoclonal antibodies or small molecule tyrosine kinase inhibitors. Each of these approaches has a distinct mechanism of action. While anti-EGFR antibodies bind to extracellular domains, the RTK inhibitors target the intracellular TK domain [102].

In melanoma, EGFR, HER3 and HER4 high expression was correlated with poor prognosis [103]. In 1985, Koprowski H. et al. reported for the first time an association between increased dosage of chromosome 7 and EGF receptor expression with melanoma progression [104] an observation confirmed in vivo [105] and in preclinical settings [106]. Consequently, studies evidenced that about 89% of primary cutaneous melanomas and 91% of melanoma metastases show a high level of either EGF or EGFR expression, suggesting them as targets for therapy [105,107].

EGFR activation during melanoma progression not only leads to the activation of various signaling pathways including PI3K/AKT and MAPK, but also promotes cell switching towards an invasive phenotype associated with loss of E-cadherin that favors release of cadherin-bound β-catenin; free β-catenin translocate to the nucleus and activate pro-invasive factors [108,109]. In addition, such activation has been also documented to cause secondary drug resistance in BRAF mutated patients under MAPK inhibitors thus opening the way to the use of EGFR inhibitors to overcome such resistance [20,110,111].

EGFR inhibitors are classified into two major groups: the first with monoclonal antibodies, such as cetuximab and panitumumab, and the second with small molecule tyrosine kinase inhibitors [112] that showed different efficacy towards melanoma cells as reported in preclinical and clinical studies. Below, we will discuss the aim and therapeutic effect of each of the tested small molecule EGFR inhibitors in melanoma.

#### 2.2.1. Gefitinib (Iressa^®^)

Gefitinib (IressaTM, ZD1839) is an anilinoquinazoline that was first FDA approved as a monotherapy in 2003 for locally advanced or metastatic NSCLC after failure of platinum based and docetaxel regimens. It is an inhibitor of intracellular tyrosine kinase activity including that of EGFR, by competitively blocking its ATP binding site [113]. A first study, carried out to check the effect of gefitinib on ErbB receptor signaling pathway in human melanoma cell lines, showed cell cycle arrest in G0/G1 phase and inhibition of cell growth shuting down PI3K/AKT, Jak/Stat, and MAPK signaling pathways (Table 6) [114]. Accordingly, a phase II study of gefitinib in patients with metastatic melanoma was conducted in 2011 and showed limited benefit, and proposed future combination strategies (Table 7) [115]. Furthermore, it was shown that gefitinib inhibits melanoma cell proliferation and invasion through the VEGF/AKT signaling pathway (Table 6) [116] and a selective inhibition of BRAF could lead to a feedback activation of EGFR that confers adaptive resistance to BRAF inhibitors in both BRAF-mutant colorectal cancer and melanoma. Therefore, simultaneous EGFR and BRAF inhibition was proposed as an effective novel combination strategy. In this context, a preclinical study evidenced that such a combination attenuates cell migration and in vivo colonization of BRAF-mutant melanoma cells (Table 6) [117].

#### 2.2.2. Erlotinib (TARCEVA^®^)

Erlotinib is a reversible, ATP-competitive inhibitor of EGFR dimerization and autophosphorylation with higher binding affinity for exon 19 deletions and exon 21 receptor mutations [125]. It showed efficacy in the treatment of non-small cell lung, colon, and pancreatic cancer and glioblastoma and was approved for the treatment of locally advanced or metastatic non-small cell lung cancer and pancreatic cancer [126].

In melanoma, as EGFR was shown to affect tumor cell functions from proliferation to differentiation as well as cell death, and as VEGF has been identified as a potent contributor to angiogenesis, tumor proliferation, and lymphangiogenesis, a first preclinical study was launched to evaluate the effect of erlotinib and bevacizumab in a human melanoma xenograft model. No effect was observed with erlotinib or bevacizumab on tumor cell proliferation, but a decreased invasive potential with erlotinib treatment in a 3D matrigel assay was shown. The combination significantly reduced angiogenesis, tumor volume and increased apoptosis (Table 6) [118]. However, a phase II trial found the combination largely ineffective in melanoma and does not merit further exploration (Table 7) [124]. Erlotinib was also combined to interleukin 24 (IL-24) based on the later expression in normal melanocytes, monocytes, and in early stages of melanoma, but was lost during progression [127,128]. IL-24 exhibits its anti-tumor activity in a broad spectrum of cancers including melanoma [129,130,131] through inhibition of PI3K, EGFR, and PKR induction in breast and NSCLC [132,133]. However, IL-24 molecular mechanisms and signaling pathways underlying melanoma suppression were not described and even less in combination with EGFR targeted therapy. Nevertheless, a preclinical study indicated a benefit from Ad-IL-24 and erlotinib in terms of tumor growth inhibition and induction of apoptosis through Apaf-1 and AKT signaling pathway inhibition (Table 6) [119].

#### 2.2.3. Lapatinib (Tyverb^®^)

Lapatinib is an oral reversible dual tyrosine kinase inhibitor that blocks EGFR and HER2, both frequently overexpressed in human cancer. Lapatinib selectively targets both EGFR and HER2 and acts in a similar way to gefitinib but in contrast to other EGFR inhibitors, can bind to an inactive form of its target [134]. Several reports indicates that ErbB and MET were found highly deregulated in melanoma patients, which made these receptors promising therapeutic targets to evaluate. Targeting each receptor alone requires administration of higher doses of the drug which often leads to acquired resistance to monotherapy along with several works indicating a crosstalk between MET and EGFR [108]. This interaction could be responsible for amplification of tumor signal transduction and receptor function compensation when only one of these receptors is inhibited. Consequently, combined therapy targeting both receptors was predicted to be effective to suppress activation of shared signal transducing pathways and crosstalk-induced positive feedback loops [120]. This combination (foretinib “MET inhibitor”and lapatinib or gefitinib) showed synergistic effect in different melanoma cells with different levels of RTK (cells express EGFR and MET) (Table 6) [120]. In continuity, and following the preclinical success of this combination in melanoma, it was tested for its efficiency on cell invasion ability and metastasis. Lapatinib alone inhibits invadopodia formation. Combining lapatinib or gefitinib with foretinib influences migration, invasion, invadopodia formation, actin cytoskeleton organization and proteolytic activity that consequently predict important combination therapeutic strategy to prevent melanoma growth and metastasize (Table 6) [108]. Moreover, hyperactivation and overexpression of RTKs were described as one of the mechanisms of acquired resistance to BRAF inhibitors through reactivation of key signaling pathways (MAPK, PI3K/AKT) and changes in the cells’ interactions with the tumor microenvironment [135]. Particularly, a hyperactivation in EGFR and MET in cells with acquired resistance to BRAF inhibitors was shown; consequently, this combination (lapatinib+foretinib) was tested for its efficiency in BRAF resistant melanoma cells. It was reported that this combination reduces cell viability and invasiveness of drug-resistant cells (Table 6) [121]. However, more benefits could be expected from irreversible EGFR-TKIs and combined treatment settings.

##### Second Generation of EGFR TK Inhibitors

Acquired resistance to the first generation of EGFR TKIs has prompted the clinical development of more potent and effective compounds with irreversible and covalent binding to the EGFR kinase domain with a broader spectrum of mutations including T790M [136]. Unlike reversible EGFR inhibitors, this generation contains an acceptor-group that binds covalently with the Cys797 at the ATP-binding site of mutant EGFR. Due to their characteristics, irreversible EGFR inhibitors seemed to be ideal to overcome T790M acquired resistance [137].

#### 2.2.4. Afatinib (Giotrif^®^)

Afatinib is an irreversible autophosphorylation inhibitor of the ErbB family of tyrosine kinases (EGFR, HER2 and HER4) [138]. It can overcome a specific resistance to EGFR inhibitors conferred by EGFR-T790M mutation in lung cancer [123]. In melanoma, afatinib was tested and, as gefitinib and lapatinib, it showed minimal cytotoxic activity alone but was more effective when combined with AKT inhibitors in vemurafenib resistant BRAF mutated melanoma (Table 6) [122]. Another combination of afatinib with crizotinib (a MET inhibitor) showed efficacy, was proposed as a promising alternative targeted therapy option for melanoma irrespective of BRAF/NRAS mutational status, and may overcome resistance to BRAF inhibitors (Table 6) [123].

### 2.3. HGFR (MET Receptor)

**Figure 3:**
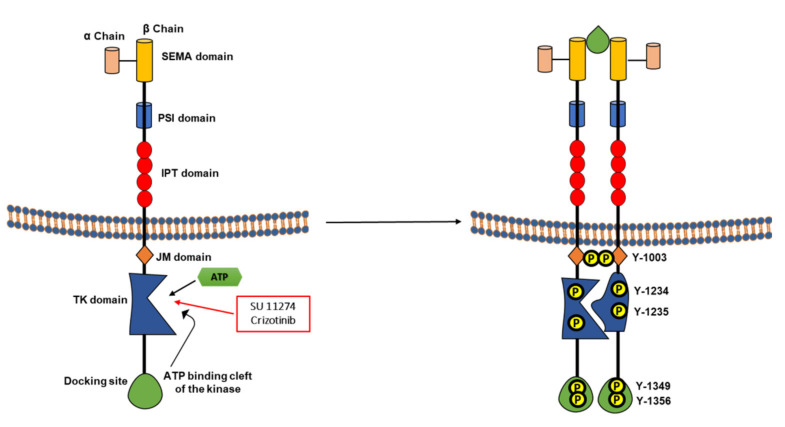
HGFR structure and activation. (**A**) ECD domain of HGFR consists of disulfide bond-linked α and β subunits, which consist of Sema, PSI, and IPT domains, followed by a transmembrane domain (TM), juxtamembrane domain (JM), tyrosine Kinase domain (TK), and docking sites for adaptor proteins. (**B**) HGF/SF in absence or presence of accessory molecule such heparin bind ECD (dimerization) that mediates autophosphorylation of specific tyrosine residues (Y1003, Y-1234, Y1235, Y1349, Y1356). HGFR-tyrosine kinase inhibitors bind the ATP-binding pocket of the kinase domain.

The hepatocyte growth factor receptor is a proto-oncogene that encodes a tyrosine kinase receptor located on chromosome 7 band 7q21–q31 and covers more than 120 kb in length, consisting of 21 exons separated by 20 introns [139]. Hepatocyte growth factor (HGF) was identified as a natural ligand for Met receptor protein [140] along with scatter factor (SF), indicating that, alone, it can transduce multiple biological processes such as motility, proliferation, survival, and morphogenesis [139]. The Met receptor is a member of a larger family of growth factor receptors sharing a similar domain architecture that includes the Ron (macrophage stimulating 1-receptor [141]) and Sea (receptors of poorly characterized biological functions [142]) receptors. HGF and HGFR are essential for normal development. In adults, both are widely expressed in several tissues, but their expression is normally very low and particularly involved mainly in tissue damage, repair, and regeneration [143]. HGFR consists of α and β chain subunits linked by a disulfide bond. The β chain consists of an extracellular domain, a transmembrane domain, and an intracellular portion. The extracellular portion of Met family members is composed of three domain types (sema, PSI, and IPT). The N-terminal (500 residues) fold into a sema domain, which form a seven-bladed β-propeller structure. The second, the PSI domain (50 residues) containing four disulfide bonds, follows the sema domain and is connected via the third segment IPT domain to the transmembrane helix and the kinase domain in the intracellular portion of the receptor. IPT domains are related to immunoglobulin-like domains (Figure 3A). The PSI domain is thought to function as a linking module to orient the extracellular fragment of Met for proper ligand binding [144]. Other reports claimed that the sema domain is the ligand binding domain of HGF [145]. Furthermore, others indicate that IPT repeats 3 and 4, found near the transmembrane domain, also serve as HGF binding [146]. Crystallographic analysis indicated that residues Thr124–Asp128 and Asp190–Phe192 in the Sema domain serve as binding interface to HGF [147,148]. The extracellular domain, shown as a rod-shaped monomer, binds HGF/SF in the absence or presence of accessory molecule heparin, and could form a complex HGF/SF–heparin–MET with a 1:1:1 stochiometry [145]. The intracellular domain of MET receptor comprises a tyrosine kinase catalytic domain delimited by juxtamembrane and carboxy-terminal sequences. Phosphorylation of tyrosine 1003 in the juxtamembrane region negatively regulates this receptor through activation of the ubiquitin ligase casitas B-lineage lymphoma (c-CBL). Following HGF binding, receptor autophosphorylation occurs on tyrosine residues Y1234 and Y1235 within the activation loop of the TK domain, inducing kinase activity, while phosphorylation on Y1349 and Y1356 in the carboxyl terminal region serves as docking site for adapters protein that transmit signals downstream (Figure 3B) [149,150]. Signaling mediators involved in this pathway include Grb2, Gab1, PI3K, phospholipase C-gamma (PLCγ), Shc, Src, Shp2, Ship1 and STAT3. Of interest, Grb2 binding to docking site through Y1356 links c-Met to the MAPK pathway that regulates the cell cycle. Met signaling cross-talked/cross-linked with other signaling downstream from several membrane receptors such as RON, EGFR and ErbB2 and could assume a mechanism of resistance for cancer progression [143]. Furthermore, it was indicated that MET major signaling is the PI3K/Akt signaling axis. The p85 subunit of PI3K can bind either directly to c-MET or indirectly through adaptor GAB1, and favor signals through AKT/protein kinase B [151]. In human malignancies, the HGF-MET pathway was found altered by several mechanisms, providing tumor cells the capacity to proliferate and disseminate. The MET gene is activated by point activating mutations in small-cell lung cancer (SCLC) [152] and renal papillary carcinomas [153]. MET protein was found overexpressed in melanoma and musculoskeletal tumors [154]. Additionally, activation of the HGF-MET pathway by overexpression and up regulation has been described as the escape resistance mechanism of tumor cells against inhibition of the EGFR, RAS-RAF-MEK, and Akt–mTOR (mammalian target of rapamycin) pathways [155,156].

In melanoma, MET is involved in melanomagenesis (melanoma initiation [157], malignant transformation of melancoyte) and progression. Its overexpression in primary lesions, indicates that it could be an important factor of aggressiveness [158]. In addition, MET gene amplification [159] and an autocrine HGF/c-Met signaling loop may be involved in melanomagenesis, but the mechanism remains unclear [160]. Furthermore, prolonged HGF stimulation favors a decrease in the intercellular adhesive molecule E-cadherin involved in the regulation of melanocyte proliferation [160]. Importantly, Met is regulated by MITF, the master transcription factor within melanocyte, and driven expression of MITF is sufficient to increase MET expression [161]. In some cases, Met activation could be associated with NRAS mutation in melanoma [162]. The release of HGF leading to HGFR activation was associated with resistance to BRAF inhibition in melanoma [163,164]. Thus, MET receptor appeared as a potential therapeutic target in melanoma [165]. Below, we will discuss different strategies tested in preclinical and clinical studies of MET inhibition alone or in combination in melanoma.

#### 2.3.1. SU 11274

SU 11274 is a pyrrole indolinone class I c-Met inhibitor selective for Y1234 and Y1235 residues that competes for the Mg-ATP complex binding pocket and is the first small compound developed to specifically inactivate Met kinase function [166]. Due to several reports indicating that MET overexpression correlates with melanoma development and invasiveness, SU11274 was tested and showed efficiency inhibiting growth, enhancing apoptosis and differentiation (Table 8) [167]. In human melanoma xenografts, it reduces tumor growth and liver colonization (Table 8) [168]. Specifically, SU11274 inhibited melanoma cell proliferation, affected cell morphology, increased tumorogenecity in vivo, and altered energetic metabolism and provided evidence for a critical glycolysis regulation in melanoma initiating cells (Table 8) [169]. Moreover, resistance to SU11274 in melanoma was accompanied with an up-regulation of WNT and mTOR signaling pathways. Accordingly, targeting mTOR and WNT pathways by everolimus and XAV939, respectively, enhanced the SU11274 effect (Table 8) [170]. However, despite promising preclinical results, this compound was not a viable clinical agent.

#### 2.3.2. Crizotinib (XALKORI^®^)

Crizotinib is a potent, orally bioavailable, ATP competitive small molecule inhibitor of the catalytic activity of c-Met and ALK kinases. It received FDA approval in non–small-cell lung cancer, and several studies showed activity in MET-amplified or mutant lung adenocarcinoma, squamous cell carcinoma, and papillary renal cell carcinoma [173]. Crizotinib was not tested for its efficiency in melanoma but was used as an agent to validate the involvement of HGF release in the resistance to mutant-BRAF inhibitors [163]. Nevertheless, preclinical and ongoing clinical studies are evaluating crizotinib in uveal melanoma to prevent metastasis by a defect in ALK gene [174] (NCT02223819).

#### 2.3.3. Tivantinib

Tivantinib (ARQ 197) is a non-ATP competitor that selectively inhibits MET. It binds to an inactive, unphosphorylated form of MET and locks it in an inactive state [175]. It showed activity in several tumor cell lines and xenograft models [176]. It is a moderator of tumor invasion and resistance to therapies that target angiogenesis [177]. In patients, tivantinib showed safety profile and anticancer activity in several tumor types [178]. As MET inhibition combined to sorafenib showed additive/synergistic effect in several cancers, a phase I trial was set to evaluate sorafenib and tivatinib combination in solid tumors with high MET activity including melanoma (Table 9) [178]. Although tivatinib was developed to inhibit MET, it recently showed an activity on microtubule polymerization indicating additional targets for this drug. As vasculogenic mimicry was reported for melanoma cells to mediate invasion and metastasis, Tivatinib was tested and found efficient in inhibiting cell viability, inducing apoptosis and reducing vasculogenic mimicry (Table 8) [171].

#### 2.3.4. PHA-665752

PHA-665752 is an ATP-competitive of the catalytic activity of the Met receptor. As NRAS mutated melanoma tumors may show aberrant c-Met activation contributing to their aggressive nature, PHA-66752 was tested in this subgroup. PHA-66752 showed a unique sensitivity in NRAS mutant melanoma in terms of cell migration inhibition and apoptosis induction (Table 8) [162].

#### 2.3.5. Quercetin

Quercetin, is a bioflavonoid found in a variety of plant-based foods such as onions, apples, tea, broccoli, and red wine. It has been reported as a potent STAT3 inhibitor in glioblastoma and gastric cancer cells [179]. Several studies indicated a significant inhibitory potential of this compound on HGF/Met signaling pathway [180]. In melanoma, Quercetin inhibited cell growth, migration and lung metastases [179]. It inhibits c-Met phosphorylation by interfering with c-Met dimerization, and consequently reduces the activities of downstream activated molecules such Gab1, FAK and PAK (Table 8) [172].

### 2.4. VEGFR

**Figure 4:**
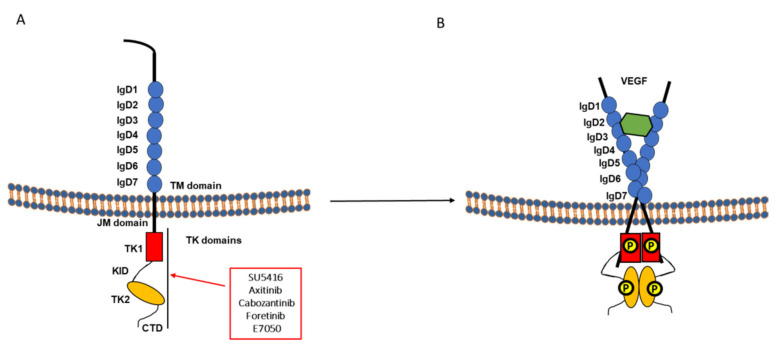
VEGFR structure and activation. (**A**) ECD of VEGFR is composed of a single peptide that consists of seven Ig-like subdomains (IgD1∼7), transmembrane domain (TM), juxtamembrane domain (JM), tyrosine kinase 1 domain (TK1) including ATP binding domain, kinase insert domain (KID), tyrosine kinase 2 domain (TK2), and a flexible C-terminal domain (CTD). (**B**) VEGFs binding to VEGFR require two Ig-like subdomains 2 and 3 (IgD2 and IgD3), and the stabilization of dimers and VEGF-mediated activity required Ig-like subdomains 4∼7 (IgD4∼7). This mediates tyrosine residues phosphorylation on the TKD and downstream signaling pathways activation. VEGFR-tyrosine kinase inhibitors occupy the ATP-binding pocket of the kinase domain to exhibit its inhibitory function.

Angiogenesis is mediated by a variety of signaling molecules; among these are the vascular endothelial growth factors (VEGFs) and receptors (VEGFRs). VEGF receptors are classified as type V RTKs. VEGFR1 is located on chromosome 13q12, VEGFR2 on chromosome 4q11-q12 [182], and the VEGFR3 gene is located on chromosome 5q35 [183]. VEGF receptors show structure homology but display interesting differences in their kinase activities and spectrum of transduced biological responses [184]. VEGFs ligands bind to three types of RTKs, VEGFR-1 (Flt-1), VEGFR-2 (KDR, Flk-1), and VEGFR-3 (Flt-4). The VEGF gene family encodes soluble glycosylated and released cytokines that form dimers: VEGF-A, VEGF-B, VEGF-C, VEGF-D, VEGF-E and placenta growth factors (PlGF-1, PlGF-2) [185]. A critical difference between VEGFR1 and VEGFR2 or VEGFR3 is that VEGF-A binds to VEGFR1 with a higher affinity, and the later shows a selective binding to VEGF-B and PlGF (placenta growth factor). Furthermore, VEGF-C and VEGF-D, highly specific for VEGFR3 and could bind to VEGF2. VEGFR2 and VEGFR3 are stronger kinases compared to VEGFR1, similar to other RTKs such as EGFR and PDGFR [2,186,187]. VEGFR-2 activates a broad signaling pathways and biological processes [188]. Both VEGFR-1 and VEGFR-2 are expressed by endothelial cells and could form heterodimers leading to autophosphorylation, activation of VEGFR-2, and angiogenesis [189]. Human VEGFR1 consists of 1338 amino acids, distributed in three major domains: an extracellular region consisting of seven immunoglobulin (Ig)-like domains, a transmembrane domain, a tyrosine kinase domain (70-amino acid residues), and a kinase insert region followed by a downstream C-terminal region. VEGFR2 (KDR in the human) composed of 1356 amino acids as well as VEGFR3 (also denoted Flt-4) are similarly organized and show 80% similarities to VEGFR1 in the tyrosine kinase domain (Figure 4A). The main difference with VEGFR3 is within the extracellular domain, by replacement of the fifth Ig-like loop by a disulfide bridge that keeps the proteolytically cleaved N-terminal part of the extracellular domain connected with the remainder of the molecule [190]. VEGFR1 and VEGFR2 ligands bind to the extracellular region to the second and third Ig-like domains and show symmetrical 2∶2 complex structure (Figure 4B) [186,191,192]. The fourth Ig-like domain appears essential for VEGFR dimerization. Analysis of the VEGF/VEGFR-2 complex indicates that a dimeric VEGF ligand binds the Ig-like domains 2 and 3 of one receptor monomer and favors the possibility of a second receptor monomer to bind the already tethered ligand (ligand mediated dimerization). Once the two receptors are cross-linked through ligand simultaneous interaction, their Ig-like domain 7s are held in close proximity to further stabilize the receptor dimers [193]. The intracellular domain contains two kinase domains named KD1 and KD2, which are split by a kinase-insert domain of 70 amino acids. Five tyrosine residues have been reported as major phosphorylation sites: Y951 (KID), Y1054 and Y1059 (ALP domain), and Y1175 and Y1214 (carboxyl terminus domain). Phosphorylation of these residues, together with the adjacent amino-acid sequence, mediate a docking site for the SH2 domains of various signaling molecules **[188,194]**. Particularly, phosphorylation of tyrosine residue 1214 plays a crucial role in the autophosphorylation and kinase activation of VEGFR-2. The intracellular regions of both VEGFR-1 and VEGFR-2 adopt a bilobal structure that is split by the kinase insert domain. The N-lobe of the kinase domain consists of antiparallel β-sheets and a single α helix denoted αC-helix. The reorientation of αC-helix is crucial to mediate the kinase switch. The active site of the enzyme is located in the cleft between the N- and the C-lobe. Activation of tyrosine kinases requires the phosphorylation of tyrosine residues on both the JMD and in the activation loop domain that causes the reorientation of αC-helix. In the open conformation, ATP and substrates bind to specific residues of the enzyme between the N- and the C-lobe. Following activation, detachment of the γ-phosphate of ATP and its transfer to the substrate occurs in the closed conformation. ADP release and phosphorylated substrate occur in the transition from the closed to the open. The JMD of VEGFR-2 may show a similar mechanism previously found in type III RTKs. It was reported that receptor dimerization is important but not sufficient for receptor kinase activation that requires conformational changes in the TMD of VEGFR-2 on glutamic acid residues. Furthermore, it was shown that ECD of VEGFR-2 plays a critical role in maintaining the receptor in the inactive state in the absence of ligand. Moreover, during the activation process, the ECD promotes the correct TMD conformation, which culminates in the proper orientation of the intracellular kinase domains to favor receptor activation [195]. In contrast to TKRs that activate the MAPK or PI3K pathways, the PLCγ-PKC-MAPK pathway is highly activated in VEGF-bound VEGFR-2 and its crucial signal for endothelial proliferation and proangiogenic signaling [196]. In melanoma, high VEGF expression was associated with poor prognosis [197]. VEGFR-1 and VEGFR-2 expression and VEGF-A release were reported in several melanoma cells [198]. Immunohistochemical analysis, indicated that VEGF is expressed by most of primary melanomas [199,200]. It was indicated that over release of VEGF and upregulated VEGFR expression favors melanoma growth through MAP kinase and PI3K signaling pathways [201]. Additionally, high mRNA and protein expression of VEGF and other pro-angiogenic mediators were associated with poor melanoma patients outcome [202,203] supported by the higher VEGF and VEGFR-2 expression in metastatic compared to primary lesions. Moreover, upregulation of VEGFR-1 was evidenced as a mechanism of resistance to the mutant-BRAF inhibitor vemurafenib in human melanoma cells [204]. Therefore, targeting VEGFR/angiogenesis could be of particular clinical importance in melanoma and splits into two groups: multikinase inhibitors (oral small molecules) or more specific monoclonal antibodies [205]. Several preclinical and clinical studies targeting VEGFR were launched but we will only focus below on the first category.

#### 2.4.1. SU5416, Sugen (Semaxanib™)

Semaxinib (SU5416, Sugen) was the first designed Flk-1/KDR tyrosine kinase inhibitor tested in several clinical trials [206]. SU5416 is a potent, ATP-competitive inhibitor of the tyrosine kinase activity of mainly VEGFR2 that showed weaker activity against PDGFR (platelet-derived growth factor receptor) and FGFR (fibroblast growth factor receptor). Early studies indicate that SU5416 was effective in A375 human melanoma mouse model when administered twice weekly [207], but a phase II trial with SU5416 as a single agent in melanoma as well as in other malignancies failed to show efficacy (Table 10) [208]. Consequently, several studies examined the benefit of dual targeting of VEGF pathway by antagonizing both VEGF production and activation. A phase II trial evaluating the efficacy and toxicity of semaxanib and thalidomide (anti-angiogenesis) combination was launched but discontinued early due to the development of other second-generation VEGFR inhibitors. However, the study underlined the feasibility, potential efficacy and the importance of future dual angiogenic targeting in melanoma patients (Table 10) [209].

#### 2.4.2. Axitinib (INLYTA^®^)

Axitinib (AG-013736) is an oral, potent, and selective second-generation inhibitor of VEGFR-1, 2, and 3, first approved in renal cell carcinoma. It showed efficacy in preclinical and clinical studies including melanoma (Table 11) [210]. A phase II melanoma trial with axitinib alone indicated its safety and efficacy in metastatic melanoma, but suggested its use in combination with other treatment modalities (Table 10) [211] Thus, axitinib enhanced OVA peptide vaccine protector activity against melanoma (Table 11) [212]. A prospective, single-arm, phase II study of axitinib with carboplatin and paclitaxel showed a safe profile and favored disease control in advanced BRAF wild-type melanoma (Table 10) [213]. Furthermore, as a consequence of poor prognosis of stage III melanoma and the promising clinical reports of anti-angiogenesis compounds, a phase II trial evaluated axitinib effect in melanoma [214]. Furthermore, encouraging studies evaluating the benefit of axitinib combination with other treatment modalities such as toripalimab (anti-PD-1) in mucosal melanoma is underway (NCT04180995) [215].

##### Dual MET/HGF and VEGF/VEGFR Targeting

Dysregulation of HGF and/or MET expression are both observed in several tumors including melanoma [176]. Additionally, angiogenesis regulated by VEGF/VEGFR axis is widely considered a crucial step in tumor progression. As result, targeting both signaling axis HGF/Met and VEGF/VEGFR could be efficient to disrupt tumorigenesis and cancer metastasis. Consequently, several TK inhibitors show dual inhibitory potential and were tested in melanoma.

#### 2.4.3. Cabozantinib (COMETRIQ^®^)

Cabozantinib (XL184) is a dual MET/VEGFR2 inhibitor acting as an ATP-competitive inhibitor of MET, VEGFR2, TIE2, and FLT3 with activity against other targets such as RET, AXL, and c-KIT. In preclinical studies, cabozantinib treatment was shown to have activity against angiogenesis and cancer progression [221]. Cabozantinib is approved as a second line treatment of medullary thyroid (MTC) and renal cell (RCC) cancers [222]. In B16F10 mouse melanoma cells, cabozantinib inhibited invasion and migration mediated by HGF (Table 12) [221]. Additionally, due to its encouraging results in phase I trials in multiple cancer types, the limited efficiency of VEGFR inhibitors as montherapy [223], the involvement of MET in the resistance to vemurafenib in BRAF mutated melanoma [163,224] and the importance of a dual targeting of MET and VEGFR, a randomized phase II trial of cabozantinib in metastatic melanoma was launched and showed clinical benefit independent of BRAF mutation status but was discontinued because it was underpowered to draw conclusions (Table 9) [181]. Cabozantinib also showed immune-modulatory effect in several cancers. Therefore, several clinical trials are ongoing to test cabozantinib with immunotherapy in melanoma [225,226] (NCT03957551, NCT04091750).

#### 2.4.4. Foretinib (Exelixis, GlaxoSmithKline) (XL-880)

Foretinib (GSK1363089) is an oral multikinase inhibitor targeting MET, RON, AXL, Tie-2, VEGFR, c-KIT, Flt-3, and PDGFR signaling pathways. It was found particularly effective against gastric and renal cancer. Foretinib, has been also used as a first-line therapy in hepatocellular carcinoma, and HER2-positive (phase I) and triple-negative breast cancer (phase II) [120]. Foretinib acts by inhibiting HGF-induced MET phosphorylation, VEGF-induced phosphorylation and precludes both HGF-mediated responses of tumor cells and HGF/VEGF-stimulation [227]. In melanoma, foretinib inhibited HGF-induced cell migration and invasion, MET phosphorylation, tumor growth and lung metastases of B16F10 model. In addition, foretinib prevented in vitro endothelial tube formation in response to VEGF, suggesting an antivascular activity (Table 12) [228]. Additionally, it significantly affected melanoma cell viability in a dose-dependent manner, changed nuclei morphology, and accumulated cells in phase G2/M (Table 6) [120]. Foretinib combination with EGFR inhibitors synergistically decreased cell viability, invasion, and migration (Table 6) [120]. Like lapatinib, foretinib alone reduces migratory capacities, invasion, Src phosphorylation and invadopodia formation in melanoma. These activities are more pronounced when combined with an EGFR inhibitor (Table 6) [108].

#### 2.4.5. E7050

E7050 (Eisai) is an oral, ATP-competitive, dual inhibitor of Met-VEGFR. It circumvented resistance to EGFR tyrosine kinase inhibitors by blocking the Met/Gab1/PI3K/Akt pathway in vitro [229]. A unique phase II clinical trial tested its combination with a VEGFR inhibitor (E7080) in advanced melanoma (NCT01433991) [230].

### 2.5. Other RTKs

#### 2.5.1. IGF1R

The type 1 insulin-like growth factor receptor (IGF1R) is a class II transmembrane receptor tyrosine kinase (RTK) that regulates key functions in cell growth and differentiation. IGF1R is broadly expressed across many cell types in fetal and postnatal tissues. Binding of the secreted growth factor ligands IGF-1 and IGF-2 to the ECD of the receptor, activates various downstream cellular responses such cell proliferation, cell death prevention or apoptosis [231]. IGF1R dysregulation has been associated with several human diseases such growth retardation and cancers [232]. Elevated levels of IGFIR are described in a variety of tumor types, and the IGF-1 axis was shown to be a predisposing factor in the development of human breast and prostate cancer. Furthermore, it was reported that IGF1R expression correlates with melanoma progression [233], although early observation indicates the absence of IGF-1 expression in melanoma cells [234]. Later, a study indicated implication of IGF-1 in melanoma pathophysiology through activation of anti-apoptotic proteins Bcl-2 and Bcl-XL and surviving [235]. Few reports discussed and evaluated IGF1R inhibition in melanoma. However, recently, it was reported that phosphatase activity of PTEN increases IGF1R expression which enhances melanoma cells resistance to vemurafenib and targeting IGF1R could be useful in melanoma patients with PTEN-positive tumors to overcome therapy resistance [236].

#### 2.5.2. FGFR

The human fibroblast growth factor receptor (FGFR) is a class IV transmembrane receptor tyrosine kinase. Similar to other RTKs, FGFRs are expressed on the cell membrane and can be activated following binding of FGF ligands to the ECD of the receptor. This mediates to FGFRs dimerization and subsequent, transautophosphorylation event of the intracellular kinase domain and activation of downstream transduction pathways, which regulates several physiological process such as proliferation, survival, differentiation, and cell migration [2,237,238]. Aberrant FGFRs expression has been shown in several solid malignancies such myeloma, bladder cancer, and non-small cell lung cancer and consequently, clinical drugs specifically targeting FGFs or FGF receptors were developed and tested in several diseases [238]. It was reported that an activating mutation of FGFR3 can augment the invasiveness of many tumors. As well, FGFR3 amplification or overexpression was shown to increase tumor progression [239]. In melanoma, FGFR1 regulates growth, angiogenesis, migration, and metastasis [240]. Additionally, FGFR2 promotes melanoma metastasis and recently, the implication of FGFR3 in melanoma growth, metastasis, and EMT behaviors was elucidated, through modulation of phosphorylation levels of ERK, AKT, and EGFR [241]. Furthermore, FGF/FGFR signaling contributes to intratumoral angiogenesis, melanoma survival and resistance to therapeutics [242]. Consequently, drugs targeting FGF/FGFR signaling are considered in combination treatment for melanoma patients showing resistance to (BRAF)/MEK inhibitors (ongoing LOGIC-2 phase II clinical trial [243]). The clinical trial testing FGFR inhibitor in melanoma is still ongoing and no published result indicates its efficiency in melanoma.

## 3. RTK Inhibitors as Immune Modulators

It was shown that RTK inhibitors may positively affect antitumor immunity. Indeed, it was reported that imatinib regulates immune cells involved in tumor immunosurveillance, boosts natural killer-cell-induced IFNα release and elicits antigen-specific T-cell responses that can prevent cancer relapses [244]. In addition, sunitinib is able to moderate regulatory T cells and thus increase T cells function [245]. In contrast, imatinib, dasatinib, or nilotinib, through off-target inhibition of kinases, can reduce memory B-cell activity that induces significant impairment of B-cell responses [246]. However, it was shown that imatinib and nilotinib exhibit variable effects on antitumor immunity, as both may impair differentiation of monocytes to DCs and reduce the activation of CD1a and CD83, but only nilotinib inhibits DC migration and consequently T-cell immune responses [247]. Thus, for future combinatory approaches, the most suitable KIT inhibitor deserves careful consideration. Additionally, EGFR inhibitors efficacy is not solely based on their direct effects on tumor cells, but also may act on the regulation of tumor microenvironment [248]. Particularly, cetuximab activates host anticancer immune response. Additionally, it was shown that EGFR inhibitors increase expression of class I and class II MHC molecules and influence adaptive immune response [249]. Furthermore, targeting EGFR could suppress T regs function, thus aiding and improving the efficacy of immunotherapy. In addition, RTK inhibitors, particularly sunitinib, pazopanib, sorafenib, and axitinib, also regulate immune effector cells activity and function. They reduce T cell proliferation, absolute neutrophils [250], monocytes [251], and lymphocyte T counts [245], causing related adverse events. Moreover, concomitant c-MET inhibition favors adoptive T cell transfer by increasing effector T cell infiltration in tumors [252]. Targeting c-Met impairs the recruitment of tumor-infiltrating neutrophils in response to immunotherapy [253]. Thus, targeting immunosuppressive cells enhances antitumor T cell response and warrants their combination with immunotherapy.

## 4. RTK Inhibitors and Check-Point Inhibitors Combination

The inhibition of PD-1 and PD-L1 axis achieved dramatic and durable responses in certain solid cancers such as melanoma, non-small cell lung cancer, and renal cell carcinoma. Combining immunotherapy with other treatment modalities is another approach to increase anti-tumor immunity through several mechanisms and is currently being tested in a number of clinical trials. In melanoma, several recent studies evaluated various targeted therapies, such as MAPK inhibitors in combination with immunotherapy, and are currently ongoing [254]. Additionally, combining EGFR antagonists to immunotherapy showed efficacy in a melanoma mouse model [248]. Interestingly, the treatment sequence is of importance as suggested by the finding that targeting angiogenesis (VEGFR inhibitor) followed by vaccination showed a better anti-tumor effect than the reverse [255]. It was also shown that treatment with anti-PD-1 nivolumab significantly prolonged OS compared to mTOR inhibitor (25.0 months vs. 19.6 months), among patients previously treated with antiangiogenic treatment. This benefit of nivolumab was observed in patients previously treated by pazopanib and not sunitinib [256], indicating that a specific targeting of VEGFR is behind such an effect. Furthermore, the HGF/MET pathway is one of the main mechanisms of resistance to anti-PD-1/PD-L1 immunotherapies, emphasizing the potential of MET-targeted therapies in PD-1/PD-L1 combinational strategies [252].

## 5. Conclusions

In BRAF-mutant melanoma, combining BRAF/MEK inhibitions and immune checkpoint blockade shows a synergistic and potentially safe response and is currently being investigated, taking drug resistance mechanisms into consideration. Conversely, in non- BRAF-mutated melanomas, novel combination strategies are still highly needed. In addition to NRAS mutations, several receptor tyrosine kinases (RTKs) such as c-KIT, EGFR, MET, and VEGFR have been reported to be involved in melanoma progression, invasion, or resistance to therapies, mainly targeting the MAPK pathway [20,121,204]. Several studies showed that BRAF V600 mutations developed resistance to BRAF or MEK inhibitors due to an up regulation of receptor tyrosine kinases such as VEGFR, MET, and/or EGFR [123]. On the other hand, up-regulation of MET and/or EGFR is one of the reported resistance mechanisms to BRAF/MEK inhibition. Consequently, the Foretinib and Afatinib combination is currently proposed in melanoma to overcome such resistance. Among c-KIT inhibitors, imatinib appears to display both efficacy and safety in melanoma. Additionally, the c-MET inhibitors crizotinib and cabozantinib showed the best clinical responses; particularly, cabozantinib as a dual MET-VEGFR inhibitor elicited particular immunomodulatory effects, making it a suitable partner for checkpoint inhibitors. Although targeting RTKs alone in melanoma did not show promise that is due to low compounds specificity, their major role in treatment escape mechanisms seems to be of a particular importance suggesting combination strategies that include RTK inhibition, particularly with check-point inhibitors. The major challenge for such an approach is depicting and understanding the associated mechanisms of action that vary from among the different melanoma subgroups.

## Figures and Tables

**Table 1:** Summary of the effect of different KIT inhibitors evaluated in melanoma preclinical studies.

RTKi	Cell Lines Used	Results	Reference
Imatinib	A375SM (from pooled lung metastases by A375 cells (^V600E^BRAF), intravenously injected to nude mice MeWo cells (^WT^BRAF, ^WT^NRAS) injected into nude mice	-Imatinib did not affect A375SM and MeWo growth in vivo but inhibits PDGFR-α and PDGFR-β phosphorylation in A375SM xenografts.	McGary, E.C., et al. 2004 [37]
Imatinib	B16F10 murine melanoma cells in C57B16 mice	-Imatinib inhibits B16F10 melanoma cell proliferation and growth in a mouse model.	Redondo, P., et al. 2004 [38]
Imatinib	M6 (^V559A^c-KIT), M40 (^WT^c-KIT), GIST 882(^K642E^c-KIT)	-Imatinib inhibits cell proliferation, MAPK, P3K/AKT, STAT pathways, favors G1 arrest, enhances apoptosis, and reduces cyclin D1, in M6 and GIST 882 cells. -Imatinib reduces BCL-2, MCL-1, ML-IAP, and survivin in both M6 and GIST882 cells.	Jiang, X., et al. 2008 [44]
Imatinib/ nilotinib	SKMel28 (^V600E^BRAF), M230 (^L576P^c-KIT), IMR_A829P (imatinib-resistant clone) NR T670I (nilotinib-resistant clone)	-M230 but not SKMel28 melanoma cells are sensitive to imatinib and nilotinib. -Drug-resistant clones exhibit secondary c-KIT mutations and retain c-KIT activation even in the presence of inhibitors. -IMR_A829P cells retains a strong apoptotic response to nilotinib and dasatinib. -NR_T670I cells undergo significant apoptosis in response to sunitinib.	Todd, J.R., et al. 2013 [55]
Nilotinib	M230 (^L576P^c-KIT)	-Nilotinib inhibits cell proliferation and reduces STAT3 signaling.	Delyon, J., et al. 2018 [56]
Dasatinib	Lox-IMVI (^V600E^BRAF), Malme-3M (^V600E^BRAF), Sk-Mel-5 (^V600E^BRAF), Sk-Mel-28 (^V600E^BRAF) and HT144 (^V600E^BRAF)	-Dasatinib inhibits growth of Lox-IMVI, Malme-3M, HT144, cell migration and invasion in HT144 and Sk-Mel-28. -Dasatinib increases apoptosis in Lox-IMVI, Malme-3M, favors G1 arrest in Lox-IMVI and HT144 and enhances response to temozolomide in HT144, Malme-3M and Lox-IMVI.	Eustace, A.J., et al. 2008 [57]
Dasatinib	MeWo (^WT^BRAF, ^WT^NRAS), SK-Mel-5 (^V600E^BRAF), SK-Mel-28 (^V600E^BRAF), A375 (^V600E^BRAF), A2058 (^V600E^BRAF), G361(^V599E^BRAF), 1205-Lu (^V600E^BRAF) 451-Lu cells (^V600E^BRAF)	-Dasatinib inhibits migration and invasion in 1205-Lu and A2058 cell lines. -Dasatinib decreases MMP9, inhibits EphA2 kinase activity and blocks SFK in A2058 cells.	Buettner, R., et al. 2008 [58]
Dasatinib	WM3211 (^L576P^c-KIT), A375 (^V600E^BRAF), MeWo (^WT^BRAF, ^WT^NRAS)	-L576P mutation induces structural changes in KIT that reduce imatinib affinity. -Dasatinib alone reduces cell viability of the L576P mutant cell line.	Woodman, S.E., et al. 2009 [59]
Dasatinib	A375(^V600E^BRAF), Sk-Mel-5 (^V600E^BRAF), Sk-Mel-28 (^V600E^BRAF)	-Dasatinib inhibits growth of melanoma cell lines and synergized with cisplatin	Homsi, J., et al., 2009 [60]
Dasatinib	Mel-p (primary melanoma), A375 (^V600E^BRAF, ^Q61K^NRAS)	-Dasatinib leads to growth inhibition of Mel-p. -Dasatinib induces cell differentiation, remodels the actin cytoskeleton, and inhibits nuclear translocation of ERK1/2	Wu, J., et al. 2013 [61]
Dasatinib	Lox-IMVI (^V600E^BRAF), Malme-3M (^V600E^BRAF), M14(^V600E^BRAF), Sk-Mel-5 (^V600E^BRAF), Sk-Mel-28 (^V600E^BRAF)	-Lox-IMV, WM-115 and HT144 cells showed sensitivity to dasatinib. -Malme-3M, WM266-4, M14, Sk-Mel-28 and Sk-Mel-5 cells were resistant to dasatinib. -High protein expression of ANXA1, CAV-1 or EphA2 in the sensitive melanoma cells that predicts sensitivity to dasatinib	Eustace, A.J., et al. 2014 [62]
Dasatinib	MDA-MB-435S, A375(^V600E^BRAF, ^Q61K^NRAS), WM853 (^V600E^BRAF)	-SIRT2 silencing renders melanoma cells more sensitive to dasatinib. -SIRT2 loss enhances dasatinib effect on cell migration inhibition and cell cycle arrest.	Karwaciak, I., et al. 2019 [63]
Sunitinib	A-07 and R-18 human melanoma cells transfected with GFP in female BALB/c nu/nu mice	-Sunitinib increases hypoxia, vessel segment length, and median vessel diameter, does not affect blood supply time (BST), vascular basement membrane, vessel tortuosity and pericyte-coverage but reduces vessel density. -Prolonged exposure, reduces tumor growth.	Gaustad, J.-V., et al. 2012 [64]
Sunitinib	Amelanotic human melanoma A-07 in female BALB/c-nu/nu mice	-Sunitinib treatment does not affect tumor growth but increases microvascular density (MVD), hypoxia, necrosis, and ADC, but reduces K trans.	Gaustad, J.-V., et al. 2013 [65]
Ponatinib	KIT^WT^, KIT^V560D^, KIT^K642E^, KIT^D816V^ PDX mice	-Ponatinib reduces cell viability, KIT, AKT, ERK phosphorylation, cell proliferation, tumor growth and induces apoptosis in the KIT mutant PDX in vitro and in vivo with high affinity to KITD816V.	Han, Y., et al. 2019 [66]

**Table 2:** Effect of imatinib alone or in combination in melanoma clinical trials.

RTKi	Phase/Year Published or Presented	Population	*N*	Dose	Survival	Response	Adverse Reactions
Imatinib	Phase II, Ugurel, S., et al. 2005 [39]	Median age of 54.2 years (range, 38.9–72.0) years	18	800 mg/day	Median OS and PFS = 3.9 and 1.9 months, respectively	No objective responses	Severe (AE)s of grade 3 and 4: Exanthema, Constipation, intestinal perforation, arterial thromboembolism, suicide attempt
Imatinib	Phase II, Wyman, K., et al. 2006 [41]	Median age of 59 years (range, 37–82) years	26	800 mg/day	Median OS and PFS = 6.5 and two months respectively	No objective responses	Grade 3 and grade 4 toxicity: Gastrointestinal toxicities, nausea and emesis
Imatinib	Phase II, Kim, K.B., et al. 2008 [42]	Median age of 58 years (range, 33–83) years	21	400 mg (twice daily)	Median OS and PFS = 7.5 and 1.4 months respectively	4 SD and 1 PR	Common toxicity of grade 3 or 4: Fatigue and oedema
Imatinib	Phase II, Carvajal, R.D., et al. 2011 [31]	Median age of 71 (range, 49–88) years	28	400 mg (twice daily)	Median OS and PFS = 10.7 and 2.8 months, respectively	2 CRs, 2 PRs, 2 transient PRs, and 5 SD	
Imatinib	Phase II trial, Guo, J., et al. 2011 [45]	Median age of 57 (range, 27–76) years	43	400 mg/d	Median OS and PFS = 15 and 9 months, respectively.	10 PRs, 13 SD and 18 showed tumor regression	Common AEs: edema, fatigue, anorexia, nausea, neutropenia, elevated AST ALT
Imatinib	Phase II trial, Hodi, F.S., et al. 2013 [32]	Median age of 65 (range, 42–84) years	25	400 mg/day	Median OS and TTP = 12.5 and 3.7 months, respectively	7 patients achieved CRs or PRs	The common reported (AE)s: nausea, fatigue, anemia, hyperglycemia, and vomiting
Imatinib + Bevacizumab (Bevax)	Phase I/II trial, Flaherty, K., et al. 2015 [48]	Median age of 63 (range, 49–86) years	23	Bevax 10 mg/kg + imatinib 400 or 600 or twice 800 mg	The median PFS = 7.7 weeks	A PR was observed in 1 patient and 7 showed SD	Common toxicities: fatigue, nausea, vomiting, edema, proteinuria, and anemia, but were not commonly severe
Imatinib + Ipilimumab (IPI)	Phase I Reilley, M.J., et al. 2017 [50]	Median age of 55 years, showing KIT positive tumour	7	400 mg imatinib (one or twice daily) +IPI 1 mg/kg/3 mg/kg) on day 1 of each 21 day/cycle		One partial response observed in one KIT -mutated melanoma patient	The common (AE)s were fatigue, nausea, vomiting, anorexia, anemia, edema, diarrhea, rash, shortness of breath, constipation, neuropathy, thrombocytopenia, and infection
Imatinib	Retrospective study, Wei, X., et al. 2019 [54]	Median age of 54 (range, 11–80) with c-KIT alterations	78	400 mg/day	Median OS and PFS = 13.1 and 4.2 months respectively	2 patients were alive without disease progression	The common (AE)s were: edema, rash, fatigue, anorexia, nausea, and neutropenia. Vomiting, psychiatric symptoms, and elevated ALT or AST, in a fraction of patients

Abbreviations: AE: adverse event; CR: complete response; OS: overall survival; PD: progressive disease; PFS: progression free survival; PR: partial response; SD: stable disease; TTP: time to progression.

**Table 3:** Nilotinib monotherapy in melanoma clinical trials.

RTKi	Phase/Year Published or Presented	Population	*N*	Dose	Survival	Response	Adverse Reactions
Nilotinib	Phase II, Cho, J.H., et al. 2012 [68]	Median age 51 (range, 37–68)	11	400 mg twice daily	Median OS and PFS = 7.7 and 2.5 months, respectively	2 PRs and 5 SD	Common AEs: alopecia, skin rash and headache
Nilotinib	Phase II, Carvajal, R.D., et al. 2015 [69]	Median age of 67 years (range, 38–85 years) in 2 cohorts: (A) refractory/shows resistance to a prior KIT inhibitor; (B) patients with brain metastases	19	400 mg twice daily	Median OS = 9.1 months and TTP = 3.3 months	In Cohort A, 2 patients achieved PRs and none observed in cohort B	Toxicity rates and patterns were similar for Cohorts A and B. The Common AEs: fatigue, low-grade musculoskeletal, gastrointestinal discomfort.
Nilotinib	Phase II, Lee, S.J., et al. 2015 [70]	Median age of 56 (range, 28–81) years	42	400 mg twice daily	Median OS and PFS = 74 and 34 weeks, respectively	1 CR and 6 PRs	Most common AEs: anemia, skin rash, liver enzyme elevation, jaundice, anorexia, fatigue, and nausea.
Nilotinib	Phase II, Guo, J., et al. 2017 [71]	Median age 65.5 (range, 20–87) years	42	400 mg twice daily	Median OS and PFS = 18 and 4.2 months, respectively	3 PRs	Rash, increased blood bilirubin, nausea, decreased appetite fatigue
Nilotinib	Phase II, Delyon, J., et al. 2018 [56]	Median age 70 (range, 62–76) years	25	400 mg twice daily	Median OS and PFS = 13.2 and 6 months, respectively	1 CR and 4 PRs	The most common AEs: fatigue, rash, increased AST/ALT or cholestasis, and nausea. Three patients had drug with drawal because of grade 3 AEs

Abbreviations: AE: adverse event; CR: complete response; OS: overall survival; PD: progressive disease; PFS: progression free survival; PR: partial response; SD: stable disease; TTP: time to progression.

**Table 4:** Dasatinib monotherapy or in combination in melanoma clinical trials.

RTKi	Phase/ Year Published or Presented	Population	*N*	Dose	Survival	Response	Adverse Reactions
Dasatinib +Dacarbazine	Phase I,Algazi, A.P., et al. 2011 [74]	Median age 62.3	50	dasatinib 70 mg dacarbazine 800 mg·m^−2^	Median OS and PFS = 40.6 and 13.4 weeks respectively	Two patients showed PRs	The most common grade 3 and 4 (AE)s were: haematological, neutropenia, anaemia, and thrombocytopenia
Dasatinib	Phase II, Kluger, H.M., et al. 2011 [75]	Median age of 64 (range, 37–84) years	39	100 mg PO BID or 70 mg PO BID	Median OS and PFS = 55 and 8 weeks respectively	Two patients showed PRs	The most common (AE)s were: fatigue, dyspnea,pleural effusion, nausea, anorexia and diarrhea
Dasatinib	Phase II,Kalinsky, K., et al. 2017 [76]	Median age of 69 (range, 41–87) years	30	70 mg orally twice daily	Median OS and PFS = 7.5 and 2.1 months respectively	4 of 22 evaluable patients had PRs	The most common (AE)s: fatigue, dyspnea, nausea, anemia, pleural effusion, neutropenia, vomiting, anorexia, hypoxia, hypertension lymphopenia, myocardial infarction

Abbreviations: AE: adverse event; OS: overall survival; PFS: progression free survival; PR: partial response.

**Table 5:** Sunitinib in melanoma clinical trials.

RTKi	Phase/ Year Published or Presented	Population	*N*	Dose	Survival	Response	Adverse Reactions
Sunitinib	Phase 2, Minor, D.R., et al. 2012 [81]	Median age 75 (range, 39–92) years	12	50 mg/d, dose modifications sequentially to 37.5 and 25 mg/d for grade III or IV toxicities.	Median survival = 6 months (patients with KIT mutations); PFS = 15 months	1 CR, 2 PRs in the 4 KIT patients and 1 PR in 6 patients with KIT amplification	The frequently observed toxicities: nausea or vomiting, skin and subcutaneous disorders, hematologic toxicity, fatigue, and hypertension
Sunitinib	Phase II study, Decoster, L., et al. 2015 [82]	Median age of 55 years	39	50 mg/d for 4 weeks, followed by 2 weeks off,	The median OS and PFS = 4.3 and 1.3 months, respectively	PRs were observed in 4 patients	The most grade 3 or 4 (AE)s were asthenia, thrombocytopenia, neutropenia, and anorexia
Sunitinib	Phase 2 trial, Bucbinder, E.I., et al. 2015 [83]	Median age of 63 (range 38–86) years	52	50 mg daily or 37.5 mg daily for 4 weeks of a 6-week cycle	The median OS and PFS = 7.7 and 3.1 months, respectively	4 patients showed PRs	The most common (AE)s were fatigue, leukopenia, thrombocytopenia, nausea, neutropenia, and diarrhea

Abbreviations: AE: adverse event; OS: overall survival; PFS: progression free survival; CR: Complete Response; PR: partial response; SD: stable disease.

**Table 6:** EGFR inhibitors in melanoma preclinical studies.

RTKi	Cell Lines Used	Results	Literature
Gefitinib	RaH3 RaH5 (both ^WT^BRAF ^WT^NRAS)	-Gefitinib exhibits a dose dependent inhibition of growth, without effect on apoptosis, favors cell arrest in G1, increased expression of p27KIP1 and reduces phosphorylation of ErbB1, ErbB2, ErbB3, ERK1/2, and AKT.	Djerf, E.A., et al. 2011 [114]
Gefitinib	A375 (^V600E^BRAF)	-Gefitinib suppressed cell proliferation, mRNA, and protein expression of VEGF and AKT, invasion and induced apoptosis.	Wan, X., et al. 2018 [116]
Gefitinib	A2058 (^V600E^BRAF), HT168-M1(^V600E^BRAF), HT199(^V600E^BRAF), WM983B (^V600E^BRAF), M24met (^61R^NRAS), MEWO (^WT^BRAF, ^WT^NRAS) A431 (squamous carcinoma cells)	-Gefitinib inhibited the activity of EGFR in HT168-M1 and WM983B. -Synergistic inhibitory effect of vemurafenib with gefitinib in BRAF mutant melanoma cells. -Gefitinib reduced cell migration in just melanoma cells expressing mutant BRAF, and inhibited in-vivo liver colonization of WM983B and HT168-M1 xenografts.	Kenessey, I., et al. 2018 [117]
Erlotinib + Bevacizumab	518A2 (^V600E^BRAF), 607B (activated Ras), Sk-Mel-28 (^V600E^BRAF), A375 (^V600E^BRAF), Mel-Juso (^Q61L^ NRAS), M24met (^61R^ NRAS), 6F (isolated from an ovaric metastasis)	-Erlotinib reduces transmigration in 518A2, M24met and SK-Mel-28 cells and increase the antiangiogenic effect of bevacizumab. -Erlotinib inhibited MEK/AKT pathways. -Erlotinib in combination with bevacizumab reduces sprout length in HUVECs conditioned with 518A2 and in cells conditioned with M24met medium. -Erlotinib and bevacizumab reduce tumor volume and proliferation, enhance apoptosis, and reduce lymph node diameter and lung metastasis in mice injected with 518A2.	Schicher, N., et al. 2009 [118]
Erlotinib + Ad-IL-24	WM35(^V600E^BRAF), WM793 (^V600E^BRAF), A375(^V600E^BRAF), MeWo (^WT^BRAF, ^WT^NRAS) (metastatic), Skmel-2 (^Q61R^NRAS), SB2 (advanced-stage), Mel-2 (metastatic, passage 6) Mel-3(metastatic, passage3), A431 (squamous carcinoma)	-Erlotinib decreased the cell viability of WM35, WM793, A375, and MeWo cells. -Co-treatment of melanoma cells with Ad-IL-24 and erlotinib decreases cell growth and enhanced apoptosis through Apaf-1 and AKT signaling pathways.	Deng, W.G., et al. 2011 [119]
Lapatinib or Gefitinib + Foretinib	A375(^V600E^BRAF), Hs294T(^WT^BRAF) WM9(^V600E^BRAF)	-Lapatinib/gefitinib shows slight effect alone but in combination with foretinib decreased melanoma cell viability in A375 and Hs294T cells. The WM9 cell was the most resistant to treatment. -Foretinib alone/with EGFR inhibitors reduced proliferation of A375, induced higher apoptosis in Hs294T than A375, and reduced pAkt and pErk levels. -Lapatinib/gefitinib does not affect cell morphology or actin cytoskeleton organization, but foretinib alone or in combination changed nuclei morphology -Foretinib alone or its combination with lapatinib/gefitinib induced G2/M cycle arrest in A375, Hs294T and WW9 cells.	Dratkiewicz, E., et al. 2018 [120]
Lapatinib or Gefitinib + Foretinib	A375(^V600E^BRAF), Hs294T (^WT^BRAF) WM9 (^V600E^BRAF)	-This combination is effective in WM9 and Hs294T, while in A375 cells, the effect was similar to foretinib alone in terms of reduction of cell migration. -This combination reduces invasion in A375 and WM9 cells but is less evident in Hs294T cells. -Foretinib alone or in combination decreases proteolytic activity.	Simiczyjew, A., et al. 2019 [108]
Lapatinib + Foretinib	A375 (^V600E^BRAF)/A375 RL and WM9 (^V600E^BRAF)/WM9 RL	-This combination or foretinib alone inhibit viability and migration in both resistant cells, especially in WM9 RL.	Dratkiewicz, E., et al. 2020 [121]
Gefitinib or Afatinib/BIBW2992 + MK-2206/GSK692094	vemurafenib-resistant YUKSI cells	-Afatinib/BIBW2992 paired with MK-2206/GSK692094 and reduced growth.	Held, M.A., et al. 2013 [122]
Afatinib + crizotinib	A375 (^V600E^BRAF), SkMel24(^V600E^BRAF), SkMel28 (^V600E^BRAF), A375PR1& A375VR4 were PLX4720 or vemurafenib-resistant sublines derived from A375, SkMel2 (^Q61R^NRAS), ESTDAB102 (^Q61R^NRAS), ESTDAB105 (^WT^BRAF/NRAS), ESTDAB138 (^WT^ BRAF/NRAS), ESTDAB140 (^WT^BRAF/NRAS) ESTDAB149 (^Q61R^NRAS)	-This combination decreased cell proliferation, colony formation, invasion, promoted cell death in distinct melanoma cells, and decreased tumor growth rate.	Das, I., et al. 2019 [123]

**Table 7:** EGFR inhibitors evaluated in different melanoma clinical trials.

RTKi	Phase/Year Published or Presented	Population	N	Dose	Survival	Response	Adverse Reactions
Gefitinib	Phase II, Patel, S.P., et al. 2011 [115]	Median age of 62.5 years, (range, 19–90) years	52	250 mg/day	Median OS and PFS = 9.7 and 1.4 months, respectively	Two PRs and 13 showed SD	Well tolerated drug, and fatigue was the only grade 3 adverse event.
Erlotinib+ Bevacizumab	Phase II trial,Mudigonda, T.V., et al. 2016 [124]	Median age of 60 years, (range, 18–65) years	28	150 mg/day of erlotinib and 10 mg/kg of bevacizumab	Median OS and PFS = 6.7 and 2 months, respectively	Two PRs and 11 showed SD	Rare grade 3–4 events: fatigue and dysarthria.

Abbreviations: AE: adverse event; OS: overall survival; PFS: progression free survival; PR: partial response; SD: stable disease.

**Table 8:** Met inhibitors in melanoma preclinical studies.

RTKi	Cell Lines Used	Results	Literature
SU11274	MM-AN, MU, PM-WK, MM-RU, MM-MC, MM-LH, and RPM-EP	-SU11274 inhibits proliferation and induces cell death in all melanoma cells expressing MET. -SU11274 induces a differentiated phenotype in MM-RU, MU, and MM-MC. -SU11274 decreases ROS and inhibits tyrosine phosphorylation of c-Met in MU melanoma cells.	Puri, N., et al. 2007 [167]
SU11274	HT168(^V600E^BRAF), HT168-M1(^V600E^BRAF), HT199 (^V600E^BRAF), WM35 (^V600E^BRAF), WM983A (^V600E^BRAF), WM983B (^V600E^BRAF), M24met (^61R^NRAS), HT168-M1 human melanoma cells in SCID-mice	-SU11274 inhibited Met phosphorylation in HT168-M1 cells. -SU11274 inhibited cells proliferation in HT168-M1, HT199, WM983B, and M24met. -SU11274 favor apoptosis and inhibit migration of HT168- M1 cells. -SU11274 inhibited intrasplenic growth and liver colonization of HT168-M1 xenograft.	Kenessey, I., et al. 2010 [168]
SU11274	M14 (^V600E^BRAF), M4Beu, A375 (^V600E^BRAF), EGFP-A375 and Rel3 (hyper metastatic variant of A375); Untreated or SU11274-treated Rel3 injected into the flank of immunodeficient mice	-SU11274 increased phosphorylation of c-Met on Tyr1349 inA375 and Rel3 cells. -SU11274 inhibits cell proliferation in all melanoma cells, changes cell morphology, mediates bioenergetic alterations, increases pluripotent stem cell proteins, phosphokinase proteome profile, tumor initiation in Rel3, and mediates in vivo tumorigenicity.	Kucerova, L., et al. 2016 [169]
SU11274	MU-P (^V600E^BRAF), RU-P, EP-P, WK-P (explant culture); MU-R and RU-R (SU11274 resistant cell lines)	-Treatment with SU11274 favors seven-fold reduction in tumor size of xenografts from RU-P melanoma cells. -SU11274 in combination with everolimus and XAV939 overcome resistance associated to c-Met inhibitor in MU-R and RU-R cells.	Etnyre, D., et al. 2014 [170]
Tivatinib	C8161 (^G464E^BRAF), WM793 (^V600E^BRAF), WM293 (^V600E^BRAF), UACC, WM278 (^V600E^BRAF)	-Tivatinib inhibits cell viability and induces apoptosis and cytotoxicity in the tested melanoma cells. -Tivatinib increases vinculin in C8161 and UACC cells, RhoC in C8161 cells and reduces zyxin and FN1 mRNA in C8161 and UACC cells. -Tivatinib decreases VM formation in C8161 and WM793 cells.	Kumar, S.R., et al. 2019 [171]
PHA-665752	A375 (^V600E^BRAF), MeWo (^WT^BRAF^WT^NRAS),SK-Mel-2 (^Q61R^NRAS), SB2 cells (NRAS mutants), WM852 (^Q61R^NRAS), 451Lu (^V600E^BRAF), WM1361A (^Q61R^NRAS), WM35 (^V600E^BRAF) WM793 (^V600E^BRAF)	-PHA-66752 favors dose dependent inhibition of MET phosphorylation in all cells and completes Akt phosphorylation inhibition in NRAS mutant cells. -IC50 of PHA-66752 was lower for NRAS mutant cells. -PHA-66752 reduces migration and induces G0/G1 cell cycle arrest and apoptosis more dramatically in NRAS mutant cells.	Chattopadhyay, C., et al. 2012 [162]
Quercetin	A375 (^V600E^BRAF), A2058 (^V600E^BRAF), SK-Mel-2 (^Q61R^NRAS) MeWo (^WT^BRAF^WT^NRAS)	-Quercetin inhibited migration, invasion, MET activation, and downstream molecules in A375 and A2058 cells. -Quercetin reduced c-Met levels through FAS inhibition in A375, A2058, MeWo, and skmel-2 cells	Cao, H., et al. 2015 [172]

**Table 9:** Clinical trials with MET inhibitor in melanoma.

RTKi	Phase/Year Published or Presented	Population	N	Dose	Survival	Response	Adverse Reactions
Tivantinib + Sorafenib	Phase I, Puzanov, I., et al. 2015 [178]	Median age of 65.1 years	19	tivantinib 360 mg BID/sorafenib 200 mg BID) or tivantinib 360 mg BID/sorafenib 400 mg BID	Median PFS = 4.9 months	1 CR, 4 patients achieved PRs	The most common AEs included: rash, diarrhea, anorexia, fatigue, alopecia, palmar plantar erythrodysaesthesia syndrome, and weight reduction
Cabozantinib	Phase II trial, Daud, A., et al. 2017 [181]	Median age of 65 (range, 30–90) years	77	100 mg daily	Median OS and PFS = 9.4 and 3.8 month, respectively	5 PRs	The most common AEs were grade 3/4: fatigue, hypertension, and abdominal pain

Abbreviations: AE: adverse event; OS: overall survival; PFS: progression free survival; PR: partial response; CR: complete response.

**Table 10:** Melanoma clinical trials with VEGFR inhibitors.

RTKi	Phase/Year Published or Presented	Study Population	N	Dose	Survival	Response	Adverse Reactions
SU5416, Semaxanib	Phase II, Kuenen, B.C., et al. 2003 [208]	Median age of 53.5 (range, 23–71) years	20	145 mg·m^−2^, twice weekly	Median OS and PFS = 107.5 and 41 days, respectively.	No Response	Main (AEs): headache, phlebitis, nausea, vomiting, anorexia, diarrhea, and asthenia
SU5416 + Thalidomide (THAL)	Phase II, Mita, M.M., et al. 2007 [209]	Median age of 58 (range, 43–71)	12	Semaxanib (145 mg·m^−2^, twice/week + THAL starting 200 mg/d	Median survival = 7.3 months	One CR and one PR	The common toxicities: deep venous, thrombosis, headache, and lower extremity edema
Axitinib	Phase II, Fruehauf, J. et al. 2011 [211]	Median age 65 (range, 30–86)	32	5 mg/twice a day	Median OS and PFS = 6.6 and 3.9 months respectively	One CR and five PRs	The most common (AE)s included fatigue, hypertension, hoarseness, diarrhea
Axitinib + carboplation+ paclitaxel	Phase II, Algazi, A.P., et al. 2015 [213]	Median age 65.4 years	36	Axitinib 5 mg PO b.i.d. + carboplatin (AUC = 5) with paclitaxel (175 mg·m^−2^)	Median OS and PFS = 14 and 8.7 months, respectively	8 patients achieved PRS	The most common (AE)s: hypertension, neutropenia, and anaemia
Bevacizumab (Bevax) + low-dose interferon alfa-2b	Phase 2, Varker, K.A., et al. 2007 [216]	Median age 57.5 (range, 28–83) years	32	Bevax (15mg/kg every 2 weeks) + low-dose IFN-α2b (1MU·m^−2^ daily)	Median OS and PFS = 10 and 3 months respectively	One PR	The most (AE)s were of grade 1/2: fatigue, anorexia, myalgia, headache, nausea, vomiting
Bevacizumab (Bevax) + high dose interferon alfa-2b	Phase II, Grignol, V.P., et al. 2011 [217]	Median age 58.4 (range, 31–79) years	25	Bevax 15 mg/kg + 5MU/m IFN-α thrice weekly	Median OS and PFS = 17 and 4.8 months respectively	6 PRs, and 5 SD	The common (AE)s were fatigue, anorexia, nausea/vomiting, fever/chills, anemia
Bevacizumab (Bevax) + paclitaxel + carboplatin	Phase II, Kim, K.B., et al.2012 [218]	Median age 60 (range, 27–85) years	143	Bevax (15 mg/kg), carboplatin (area under the curve, 5) plus paclitaxel (175 mg·m^−2^)	Median OS and PFS = 12.3 and 5.6 months, respectively	3 CRs and 33 PRs	The most common (AE)s: neutropenia, peripheral neuropathy, febrile neutropenia, arterial thromboembolic events, and hypertension
Bevacizumab	Phase III Corrie, P.G., et al. 2018 [219]	Median age 56 years (18–88 years)	671	7.5 mg/kg	Median DFI = 63 months	Adjuvant bevacizumab improved DFI but did not improve OS	Common grade 3 or 4 adverse events was hypertension

Abbreviations: AE: adverse event; DFS: disease-free interval; OS: overall survival; PFS: progression free survival; PR: partial response; SD: stable disease.

**Table 11:** Preclinical studies evaluating VEGFR inhibitors in melanoma.

RTKi	Cell Lines Used	Results	Literature
Axitinib	M24met (^61R^NRAS), A375 (^V600E^BRAF) A2058 (^V600E^BRAF)	-Axitinib inhibited VEGFR-2 phosphorylation and partially ERK1/2 signal in angiogenic vessels of xenograft tumors of M24 met. -Axitinib in combination with bevacizumab inhibited lymph node metastasis and prolonged survival.	Hu-Lowe, D.D., et al. 2008 [210]
Axitinib + OVA peptide-based vaccination	MO5 (B16.OVA)	-Axitinib enhances vaccine effect to prevent melanoma growth and to favor T cell infiltration and activity. -This combination prevents accumulation of MDSC and Treg suppressor cells and promotes type-1 LT cells function in vivo.	Bose, A., et al. 2012 [212]
Bevacizumab	A-07 and D-12 meningeal human melanoma cells inoculated in the intracranial region of BALB/c nu/nu mice	-Bevacizumab inhibits angiogenesis and, increases cerebral invasion and genes related angiogenesis expression of A-07 tumors	Simonsen, T.G., et al. 2020 [220]

**Table 12:** Dual VEGFR/MET inhibition in melanoma preclinical studies.

RTKi	Cell Lines Used	Results	Literature
Cabozantinib	Murine B16F10 cells	-Cabozantinib inhibits cell tubule formation, migration, and invasion	Yakes F et al. 2011 [221]
Foretinib	Murine B16F10 cells	-Foretinib inhibits migration, invasion, and anchorage dependent growth. -Foretinib inhibits phosphorylation of Met, Flk-1/KDR, and reduces tumor burden injected in mice.	Qian F et al. 2009 [228]
